# *Panax notoginseng* WRKY Transcription Factor 9 Is a Positive Regulator in Responding to Root Rot Pathogen *Fusarium solani*

**DOI:** 10.3389/fpls.2022.930644

**Published:** 2022-07-14

**Authors:** Lilei Zheng, Bingling Qiu, Linlin Su, Hanlin Wang, Xiuming Cui, Feng Ge, Diqiu Liu

**Affiliations:** ^1^Faculty of Life Science and Technology, Kunming University of Science and Technology, Kunming, China; ^2^Yunnan Provincial Key Laboratory of Panax Notoginseng, Kunming, China

**Keywords:** jasmonic acid, *Fusarium solani*, WRKY transcription factors, transgenic tobacco, transcriptional activation

## Abstract

*Panax notoginseng* (Burk) F.H. Chen is a rare and valuable Chinese herb, but root rot mainly caused by *Fusarium solani* severely affects the yield and quality of *P. notoginseng* herbal materials. In this study, we isolated 30 *P. notoginseng* WRKY transcription factors (TFs), which were divided into three groups (I, II, and III) on the basis of a phylogenetic analysis. The expression levels of 10 *WRKY* genes, including *PnWRKY9*, in *P. notoginseng* roots increased in response to a methyl jasmonate (MeJA) treatment and the following *F. solani* infection. Additionally, PnWRKY9 was functionally characterized. The PnWRKY9 protein was localized to the nucleus. The overexpression of *PnWRKY9* in tobacco (*Nicotiana tabacum*) considerably increased the resistance to *F. solani*, whereas an RNAi-mediated decrease in the *PnWRKY9* expression level in *P. notoginseng* leaves increased the susceptibility to *F. solani*. The RNA sequencing and hormone content analyses of *PnWRKY9*-overexpression tobacco revealed that PnWRKY9 and the jasmonic acid (JA) signaling pathway synergistically enhance disease resistance. The PnWRKY9 recombinant protein was observed to bind specifically to the W-box sequence in the promoter of a JA-responsive and *F. solani* resistance-related defensin gene (*PnDEFL1*). A yeast one-hybrid assay indicated that PnWRKY9 can activate the transcription of *PnDEFL1*. Furthermore, a co-expression assay in tobacco using β-glucuronidase (GUS) as a reporter further verified that PnWRKY9 positively regulates *PnDEFL1* expression. Overall, in this study, we identified *P. notoginseng* WRKY TFs and demonstrated that PnWRKY9 positively affects plant defenses against the root rot pathogen. The data presented herein provide researchers with fundamental information regarding the regulatory mechanism mediating the coordinated activities of WRKY TFs and the JA signaling pathway in *P. notoginseng* responses to the root rot pathogen.

## Introduction

*Panax notoginseng* (Burk) F.H. Chen is a high-value medicinal herb in the family Araliaceae. The main active substances in *P. notoginseng* are triterpene saponins, which have many effects (e.g., hemostatic, anti-platelet, thrombolytic, anti-hypertensive, and anti-inflammatory effects) (Xie et al., 2018). *Panax notoginseng* plants must be cultivated under shade and humid conditions for at least 3 years before they can be used for medicine. Because of the specific environmental conditions required during its cultivation and its long growth cycle, *P. notoginseng* is susceptible to many diseases, especially root rot, which is mainly caused by *Fusarium solani* (Liu D. et al., 2019). *Fusarium solani* is a necrotrophic fungus widely distributed in soil. After conidia germination in soil, *F. solani* invades the plant host through the root tip or wound, then multiplies in roots and further spreads to the whole plant (Caroline and Olubukola, 2013). In addition, the chlamydospores of *F. solani* are formed when plants die, and these spores are released back into the soil, where they can remain viable for up to 30 years (Schollenberger et al., 2005). Root rot initially results in the decaying of *P. notoginseng* roots (i.e., main medicinal tissue), and it may eventually lead to the death of susceptible plants (Ma et al., 2019). Root rot seriously affects the yield and quality of *P. notoginseng* herbal materials. Additionally, the agricultural chemical treatments used to control root rot might make raw *P. notoginseng* herbal materials unsafe for human consumption (Hao et al., 2019; Khaliq et al., 2020). Therefore, the mechanism underlying *P. notoginseng* defenses against root rot must be clarified to enable the development of root rot-resistant cultivars.

Plant defense responses can be divided into the following two interrelated branches: pathogen-associated molecular pattern-triggered immunity (PTI) and effector-triggered immunity (ETI) (Li et al., 2021b). The signaling associated with PTI and ETI leads to a large-scale transcriptional reprogramming, which is regulated by many transcription factors (TFs). WRKY TFs are specific in plants, and have an important role in resistance. Some WRKY TFs promote susceptibility to a few pathogens while others promote resistance (Jiang J. et al., 2017). A total of 62 *WRKY* genes were identified in the pepper (*Capsicum annuum*) genome, one of which, *CaWRKY22*, positively regulated pepper resistance to *Ralstonia Solanacearum*, but *CaWRKY40b* was a negative regulator of resistance (Hussain et al., 2018; Ifnan Khan et al., 2018). Fifty-six possible *WRKY* genes in melon (*Cucumis melo*) were detected after a protein database was screened for conserved WRKY domains (Jiao et al., 2018). *CmWRKY6/31/47/48/55* positively responded to powdery mildew pathogen (*Erysiphe cichoracearum*) infection, while *CmWRKY15* was depressed during defense response. More than 50 *WRKY* genes were identified in rose (*Rosa chinensis*), and there were 19 *RcWRKY* genes may regulate rose resistance against *Botrytis cinerea* infection (Liu X. et al., 2019).

The WRKY TFs have some conserved structures associated with their transcriptional regulatory functions, including nuclear localization signals, leucine zippers, and WRKY domains. The WRKY domain comprises a heptapeptide sequence (WRKYGQK) that is usually located in the N-terminal region, whereas the zinc finger domain is located at the C-terminal (Xu et al., 2020). The WRKY proteins are phylogenetically classified in three major groups (Groups I–III) according to the number of WRKY domains and the structure of the zinc finger motif. Both of these motifs are necessary for the interaction between WRKY TFs and the W-box (TGACT/C) *cis*-element present in the promoters of many defense-related genes (Hussain et al., 2018). In apple (*Malus* × *domestica*), WRKY31 binds specifically to W-box2 in the *MdhIR4* promoter to inhibit gene expression and enhance the resistance to *Botryosphaeria dothidea* (Zhao et al., 2019a).

Plant stress hormones play an important role in response to abiotic and biotic stress (Afrin et al., 2015). The resistant soybean (*Glycine max*) had higher level of salicylic acid (SA) during *F. solani* infection than the susceptible (Bawa et al., 2019). The jasmonic acid (JA) content in peony (*Paeonia lactiflora*) leaves gradually increased, while the SA content significantly decreased after inoculation with *Alternaria tenuissima* (Wang X. et al., 2019). Our previous study demonstrated that the defense priming in *P. notoginseng* through exogenous application of methyl jasmonate (MeJA) evidently induced the resistance to root rot pathogen *F. solani* (Liu D. et al., 2019). Moreover, the JA-responsive *defensin like protein* (*PnDEFL1*) and *snakin* (*PnSN1*) act as antimicrobial peptide to enhance the *P. notoginseng* resistance to *F. solani* (Wang Q. et al., 2019; Qiu et al., 2020). These findings imply that JA contributes to resistance of *P. notoginseng* to root rot. Under stressful conditions, JA is induced to synthesize and binds to the F-box protein coronatine insensitive 1 receptor. Subsequently, the promoted degradation of JAZ transcriptional repressors by ubiquitination releases a variety of TFs, and then leads to the activation of their target genes, which finally cope with and adapt to multiple stress (Zhou and Memelink, 2016). In addition, JA often forms a crosstalk network with ethylene (ET) to respond the necrotrophic pathogens. JAZ can inhibit the activity of ethylene-stabilized transcription factors EIL2/EIN3 in the ET signaling pathway, and then activates the expression of *ETHYLENE RESPONSE FACTOR 1*, thereby resisting the necrotrophic pathogen *Botrytis cinerea* (Zhu et al., 2011). Overexpression of *ETHYLENE RESPONSE FACTOR 96* in *Arabidopsis thaliana* up-regulated expression of JA/ET-responsive defense genes including *PDF1.2a, PR-3, PR-4*, and *VSP2*, and then enhanced resistance to *B. cinerea* (Catinot et al., 2015).

Previous research revealed that WRKY TFs usually function cooperatively with plant hormone signal transduction pathways during responses to biotic stresses, including pathways involving JA, ET, SA, and abscisic acid (ABA) (Jiang J. et al., 2017). The expression of lotus (*Nelumbo nucifera*) *WRKY* genes in roots is regulated by SA and JA treatments, with the *NnWRKY40a* and *NnWRKY40b* expression levels significantly induced by JA, in particular (Li et al., 2019). In addition, WRKY75 regulated the JA-mediated signaling pathway to modulate defense responses in *Arabidopsis thaliana*. Under normal conditions, WRKY75 interacted with JAZ to inhibit the JA signaling pathway. When *A. thaliana* plants were infected by *Botrytis cinerea*, JA biosynthesis was boosted, and JAZ was degraded. Subsequently, WRKY75 was released to activate the expression of its target gene *ORA59* for enhancing disease resistance (Chen et al., 2021).

The *WRKY* genes have been widely studied in recent years, but the *P. notoginseng WRKY* gene family remains relatively uncharacterized. It remains unclear whether WRKY TFs help regulate the *P. notoginseng* defense response to root rot. Thus, in this study, the recently generated RNA sequencing (RNA-seq) data for *P. notoginseng* plants infected with *F. solani* (Liu D. et al., 2019) were used to isolate and identify *WRKY* gene transcripts. Moreover, exogenous MeJA treatment induced resistance of *P. notogisneng* to *F. solani* (Liu D. et al., 2019), the *WRKY* gene expression patterns resulting from the independent MeJA treatment and *F. solani* infection as well as the combined MeJA treatment and *F. solani* inoculation were examined on the basis of quantitative real-time PCR (qRT-PCR) analyses. To elucidate the transcriptional regulatory mechanism mediating *P. notoginseng* responses to root rot, a JA-responsive gene (*PnWRKY9*) was selected for an analysis of its function during an *F. solani* infection. Furthermore, the promoter of an *F. solani* resistance-related gene (*PnDEFL1*) was cloned and the activation of its transcription by PnWRKY9 was verified.

## Materials and Methods

### Plant and Fungal Materials

The unigenes with an open reading frame (ORF) encoding a WRKY protein were obtained by analyzing the *P. notoginseng* transcriptome sequencing data from our previous study (Liu D. et al., 2019). The susceptible 1-year-old *P. notoginseng* plants grown in a greenhouse under shading nets were used for the cloning of *WRKY* transcripts ORF and the subsequent gene expression analysis. The *F. solani* isolate used in this study, which was obtained from *P. notoginseng* plants exhibiting typical root rot symptoms, was stored at 4°C in our laboratory. After being activated on potato dextrose agar medium, it was used in the following experiments. The susceptible wild-type (WT) tobacco (*Nicotiana tabacum*) seeds used for genetic transformations were surface-sterilized in 75% alcohol and a 0.1% HgCl_2_ solution prior to sowing on 1/4 Murashige and Skoog culture medium.

### Identification of *P. notoginseng WRKY* Genes and Bioinformatics Analysis

Total RNA of *P. notoginseng* roots under normal conditions was extracted using the TRIGene kit (GeneStar, China), and then cDNA was synthesized via reverse transcription using the Eastep^®^ RT Master Mix kit (Promega, USA). Specific primers were designed for the RT-PCR amplification of *P. notoginseng WRKY* transcripts from cDNA. The PCR products were sequenced after T-A cloning to verify the accuracy of *WRKY* unigene sequences. Moreover, a phylogenetic tree was constructed using the MEGA 6.0 software. The PnWRKY9 (OM273051) protein served as the query for a BLAST search of the NCBI database (https://www.ncbi.nlm.nih.gov/). Several WRKY protein sequences highly homologous to PnWRKY9 were included in a multiple sequence alignment using the DNAMAN software to identify their conserved domains. Photoshop was used to visualize the structure of PnWRKY9. The subcellular localization of PnWRKY9 was predicted using the PredictProtein online program (http://www.predictprotein.org/).

### qRT-PCR

To analyse whether these *WRKYs* responded to JA signaling and *F. solani*, the roots of *P. notoginseng* plants were wounded with sterile scissors and then immersed in 100 μM MeJA solution (Solarbio, China) and *F. solani* spore solution (10^6^ spores/mL) for 30 min, respectively. The roots were collected at 24, 48, and 72 h post treatment or inoculation, and the roots treated with sterile water for 30 min were used as control. In addition, to investigate whether *P. notoginseng WRKYs* cooperated with JA signaling in response to *F. solani* infection, the *P. notoginseng* roots were wounded with a small pair of scissors and then immersed in 100 μM MeJA solution for 30 min. At 24 h post treatment with MeJA, these *P. notoginseng* roots were further inoculated with *F. solani* spore solution for 30 min, and then were collected at 12, 48, and 72 h post-inoculation (hpi). Meanwhile, the roots only treated with sterile water were collected as the control.

To eliminate differences among individual plants, the roots of three similarly treated or inoculated *P. notoginseng* plants were collected and combined for the qRT-PCR analysis. Total RNA was extracted from the root samples using the TRIGene kit (GeneStar, China) and then cDNA was synthesized via reverse transcription using the Eastep^®^ RT Master Mix kit (Promega, USA). Primers designed to anneal to specific *P. notoginseng WRKY* transcripts were used for the qRT-PCR analysis (Supplementary Table 1). The *P. notoginseng* actin gene (*PnACT2*, KF815706.1) was selected as the internal reference gene. The qRT-PCR analysis was completed using the Eastep^®^ qRT-PCR Master Mix kit (Promega, USA). The *PnWRKY* expression levels were calculated according to the 2^−ΔΔCt^ method.

### Subcellular Localization

Gene-specific primers (Supplementary Table 2) were designed to clone the *PnWRKY9* ORF (without the stop codon), which was then inserted into the pGEM-T^®^ Easy vector (Promega, USA). The pGEM-T Easy-*PnWRKY9* recombinant plasmid was digested with *Bam*HI and *Xba*I and the *PnWRKY9* sequence was inserted into the pBIN m-gfp5-ER vector for the subsequent expression of the PnWRKY9-green fluorescent protein (GFP). The pBIN m-gfp5-ER-*PnWRKY9* recombinant plasmid was inserted into *Agrobacterium tumefaciens* EHA105 cells according to a CaCl_2_ freeze–thaw method (Holsters et al., 1978). The *A. tumefaciens* positive clones, which were identified on the basis of a PCR amplification, were used to infect onion (*Allium cepa*) inner epidermal cells. The green fluorescence of the PnWRKY9-GFP fusion protein was detected using a confocal laser scanning microscope (Nikon, Japan). The cells were then stained with propidium iodide (PI), after which the subcellular localization of PnWRKY9 was determined on the basis of the observed fluorescence.

### Development and Analysis of *PnWRKY9*-Overexpression (OE) Tobacco

Gene-specific primers (Supplementary Table 2) were designed to clone *PnWRKY9* from pGEM-T-*PnWRKY9*. Next, the pCAMBIA2300s-*PnWRKY9* recombinant plasmid was constructed via a double digestion with *Eco*RI and *Bam*HI and a ligation mediated by T4 DNA ligase. The pCAMBIA2300s-*PnWRKY9* recombinant plasmid was inserted into *A. tumefaciens* LBA4404 cells according to the CaCl_2_ freeze–thaw method. The *A. tumefaciens* positive clones were used for the transformation of WT tobacco plants (Horsch et al., 1985). Genomic DNA was extracted from transgenic tobacco plants using cetyltrimethylammonium bromide as previously described (Allen et al., 2006). The positive transgenic tobacco plants were identified on the basis of a PCR amplification. The T_0_ generation tobacco plants were grown in a greenhouse to obtain the T_2_ generation lines for further analyses.

To analyse *PnWRKY9* transcripts levels, total RNA was extracted from T_2_ transgenic tobacco lines and used to synthesize cDNA as described above. The gene-specific primers were listed in Supplementary Table 1. The resistance of T_2_ *PnWRKY9*-OE transgenic tobacco plants to *F. solani* was evaluated after inoculating the roots. Specifically, the roots of WT and transgenic tobacco plants were injured and then soaked in an *F. solani* spore solution (2 × 10^6^ spores/mL) for 30 min. The plants were subsequently maintained in an incubator at 25°C with a 16-h photoperiod for 7 days. The disease symptoms at 7 days post-inoculation were recorded using a digital camera (Nikon, Japan).

### RNA Interference (RNAi)

Gene-specific primers (Supplementary Table 2) with attB linkers (F: 5′-GGGGACAAGTTTGTACAAAAAAGCAGGCT-3′; R: 5′-GGGGACCACTTTGTACAAGAAAGCTGGGT-3′) were used for the PCR amplification performed to obtain a *PnWRKY9* RNAi fragment. The pHellsgate2-*PnWRKY9* recombinant plasmid was constructed using the Gateway BP Clonase™ II Enzyme Mix kit (Invitrogen, USA). The pHellsgate2-*PnWRKY9* recombinant plasmid and the empty pHellsgate2 vector were inserted into separate *A. tumefaciens* EHA105 cells. Fresh *P. notoginseng* leaves with similarly sized wounds were inoculated with 100 μL solutions of *A. tumefaciens* cells containing the pHellsgate2-*PnWRKY9* recombinant vector or the empty pHellsgate2 vector (control). The leaves were placed in a foam box containing moistened filter paper and kept in an incubator for 24 h before being inoculated with *F. solani* (2 × 10^6^ spores/mL). The disease symptoms after a 72-h incubation were recorded using a digital camera. The diseased area on leaves was calculated using the Photoshop software. Additionally, total RNA was extracted from the *P. notoginseng* leaves for an examination of *PnWRKY9* expression in a qRT-PCR assay.

### RNA-Seq Analysis and Hormone Content Determination of *PnWRKY9*-OE Tobacco

High-quality total RNA was extracted from the *PnWRKY9*-OE tobacco leaves (line 9-11) and WT tobacco leaves using the TRIzol^®^ reagent according to the manufacturer's instructions (Invitrogen, USA). Genomic DNA was removed using DNase I (Takara, Japan) prior to the RNA-seq analysis, which was completed using the Illumina platform (Majorbio, China). The following data processing and analysis steps were performed using the online platform of Majorbio Cloud Platform (www.majorbio.com): generation of clean reads, alignment with the reference genome, expression analysis, identification of differentially expressed genes (DEGs), gene ontology (GO) functional annotation, and kyoto encyclopedia of genes and genomes (KEGG) pathway analysis. The RNA-seq analysis involved three biological replicates.

The JA and SA contents in the *PnWRKY9*-OE tobacco and WT tobacco plants were determined using an ultra-high performance liquid chromatography–mass spectrometry (UPLC-MS) system. Briefly, tobacco leaves (50 mg) were collected from two *PnWRKY9*-OE tobacco lines 9-3/11 and WT tobacco, respectively. Three biological replicates were included in this experiment. Samples were processed and their hormone contents were determined as described by Qiu et al. (2020).

### Cloning and Analysis of the *PnDEFL1* Promoter

In *P. notoginseng, PnDEFL1* is a JA-responsive defensin gene that mediates the resistance to *F. solani* (Wang Q. et al., 2019). The *PnDEFL1* promoter (PPnDEFL1) was amplified via genome walking for an analysis of the transcriptional regulation of *PnDEFL1* by PnWRKY9. According to the *PnDEFL1* (MK238492.1) sequence, two gene-specific primers (GSP1 and GSP2; Supplementary Table 2) were designed. The PPnDEFL1 sequence was cloned according to the Universal GenomeWalker 2.0 user manual (Takara, Japan). The putative *cis*-elements in PPnDEFL1 were predicted using the PlantCARE online tool (http://bioinformatics.psb.ugent.be/webtools/plantcare/html/).

### Electrophoretic Mobility Shift Assay (EMSA)

A pET32a plasmid expressing recombinant PnWRKY9 with N-terminal His-tag was constructed using the ClonExpress II One Step Cloning Kit (Vazyme, China) to produce the PnWRKY9 recombinant protein for EMSA. Gene-specific primers were designed and used to amplify the *PnWRKY9* ORF (without the stop codon) (Supplementary Table 2). The pET32a vector was linearized via a digestion with *Bam*HI and *Eco*RV before incorporating the *PnWRKY9* ORF. The pET32a-*PnWRKY9* recombinant plasmid was inserted into *Escherichia coli* BL21 (DE3) cells (Tsingke, China). The production of the PnWRKY9 recombinant protein was induced by adding 0.5 mM isopropyl-β-D-1-thiogalactopyranoside to the bacterial solution, which were then cultured for 6 h at 28°C. The proteins in inclusion bodies were denatured and renatured as described by Li et al. (2021b). The denatured recombinant protein was purified and concentrated using the Ni-NTA Sepharose column (Sangon Biotech, China) and the Amicon^®^ Ultra-15 centrifugal filter device (Merck Millipore, USA), respectively.

The PPnDEFL1 sequence was used to design probes (Supplementary Table 2) containing a W-box or a mutated W-box. The probes were synthesized and labeled with biotin (Sangon Biotech, China). The free probes used for the EMSA were the DNA fragments containing a W-box labeled with biotin, whereas the competitor probes lacked biotin and were used at 50-fold higher concentrations than the free probes. Additionally, the mutant probes contained a mutated W-box and were labeled with biotin. The EMSA was performed according to the standard procedure of the LightShift™ Chemiluminescence EMSA kit (Pierce, USA).

### Yeast One-Hybrid (Y1H) Assay

Genomic DNA extracted from *P. notoginseng* plants served as the template for cloning the PPnDEFL1 sequence using a pair of primers (Supplementary Table 2). The PPnDEFL1 sequence was inserted into the pAbAi yeast bait vector (Clontech, USA) that had been digested with *Bam*HI and *Hin*dIII. The PPnDEFL1-pAbAi recombinant plasmid was inserted into Y1Hgold yeast cells, which were then grown on SD/–Ura medium. The *PnWRKY9* ORF was amplified by PCR using specific primers (Supplementary Table 2) and then inserted into the pGADT7 yeast prey vector (Clontech, USA). The resulting pGADT7-*PnWRKY9* recombinant plasmid was inserted into yeast cells containing PPnDEFL1-pAbAi. The p53-pAbAi yeast bait vector and the pGADT7-*P53* yeast prey vector were from the Matchmaker^®^ Gold Yeast One-Hybrid Library Screening System kit (Clontech, USA). The positive transformants on SD/–Leu medium were identified on the basis of a PCR amplification and then transferred to SD/–Leu medium containing 200 ng/mL aureobasidin A (AbA) to analyze the potential interaction between *PnDEFL1* promoter and PnWRKY9 protein.

### Co-expression of the *PnDEFL1* Promoter and *PnWRKY9* in Tobacco

The PPnDEFL1 sequence was amplified from *P. notoginseng* genomic DNA by PCR using specific primers (Supplementary Table 2) and then inserted into the pBI121 vector to drive the expression of reporter gene (*GUS*). Moreover, the pBI121-*GUS* recombinant plasmid was inserted into *A. tumefaciens* LBA4404 cells, which were then used to infect WT tobacco and *PnWRKY9*-OE tobacco according to the leaf disk transformation method as described by Horsch et al. (1985). Finally, the infected leaf discs were kept in an incubator (25°C, 16-h photoperiod) to regenerate tobacco seedlings. The PPnDEFL1-*GUS/PnWRKY9*-OE tobacco plants and the PPnDEFL1-*GUS* transgenic tobacco plants were identified on the basis of a PCR amplification. Total RNA was extracted from both types of transgenic tobacco and then cDNA was synthesized using the Eastep^®^ RT Master Mix kit (Promega, USA). Primers specific for the *GUS* (Supplementary Table 1) were used for the qRT-PCR analysis of *GUS* expression levels, with the tobacco actin gene (*NtACT*, AB158612.1) selected as an internal reference gene. The *GUS* relative expression level was calculated according to the 2^−ΔΔCt^ method. The significance of any differences in expression was determined on the basis of Student's *t-*test, which was conducted using the SPSS software. Furthermore, GUS activities in the PPnDEFL1-*GUS/PnWRKY9*-OE and PPnDEFL1-*GUS* transgenic tobacco plants were determined using a fluorescence spectrophotometer as described by Chen R. et al. (2017).

## Results

### Identification and Bioinformatics Analysis of *P. notoginseng WRKY* Family Genes

**Figure 1:**
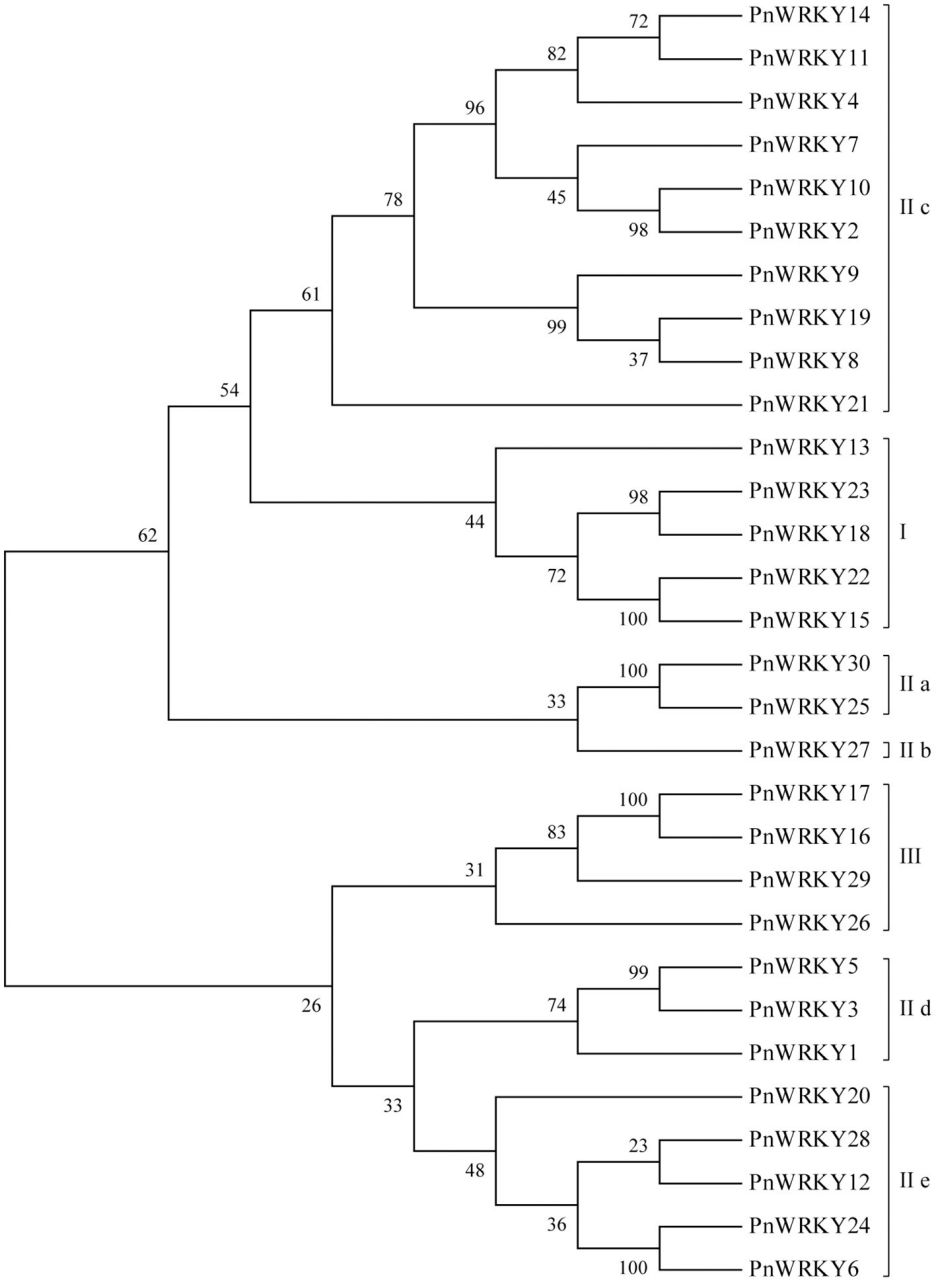
Phylogenetic analysis of the *Panax notoginseng* WRKY family. The phylogenetic tree of *P. notoginseng* WRKY family was completed with MEGA6 software. The PnWRKYs were divided into groups I, II and III, in which the group II was further divided into five subgroups (IIa, IIb, IIc, IId, and IIe).

Thirty unigenes encoding putative WRKYs were identified following an analysis of published *P. notoginseng* transcriptome sequencing data (Liu D. et al., 2019). Additionally, the full-length cDNA sequences were amplified, after which a sequencing analysis confirmed the accuracy of these *WRKY* genes, which were named *PnWRKY1–PnWRKY30* (KM925138, OM273030-OM273058). The phylogenetic tree constructed for the *P. notoginseng WRKY* gene family using MEGA 6.0 (Figure 1) revealed the WRKY TFs could be divided into three groups (I, II, and III). The Group I WRKY TFs, which contained two WRKY motifs and one C_2_-H_2_(C-X_4−5_-CX_22−23_-H-X_1_-H) zinc finger motif, included five PnWRKYs (PnWRKY13, 15, 18, 22, and 23). The Group II members had the same zinc finger motif as the Group I WRKY TFs, but they contained only one WRKY motif. Most of the 30 PnWRKYs belonged to Group II, which was further divided into five subgroups (IIa, IIb, IIc, IId, and IIe). The Group III WRKY TFs, which had a WRKY motif and a zinc finger motif [C_2_-HC(C-X_7_-C-X_23_-H-X-C)] that differed from the one in the WRKY TFs in Groups I and II, included four PnWRKYs (PnWRKY16, 17, 26, and 29).

### Several *P. notoginseng WRKY* Genes Were Responsive to the MeJA Treatment and the *F. solani* Infection

**Figure 2:**
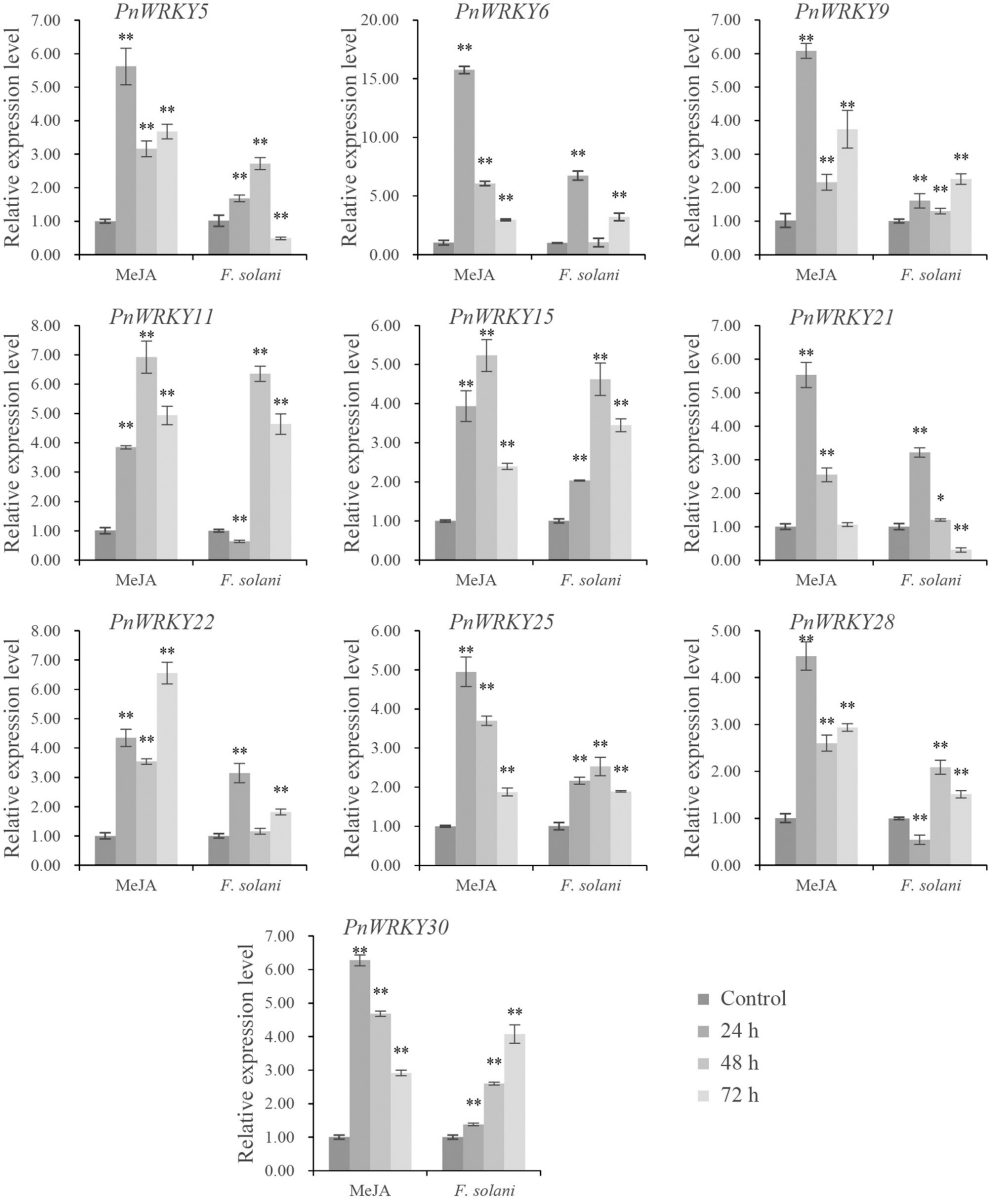
The expression levels of *PnWRKYs* during independent MeJA treatment or *Fusarium solani* infection. *P. notoginseng* roots were treated with MeJA and inoculated with *F. solani*, respectively. The roots were collected after treatment or inoculation for 24, 48, and 72 h. *P. notoginseng* roots treated with sterile water for 30 min were harvested as control. The 2^−ΔΔCt^ method and *t* test (*p < 0.05, **p < 0.01) were used to calculate the relative gene expression levels and statistical difference.

The expression patterns of some *PnWRKY* genes following the MeJA treatment and the *F. solani* infection were analyzed by qRT-PCR. The results revealed that the expression levels of several *P. notoginseng WRKY* genes increased after the MeJA treatment (Figure 2). Among these induced genes, *PnWRKY5*/*6*/*9*/*21*/*25*/*28/30* expression levels were maximum at 24 h post-treatment with MeJA. Moreover, the transcription of these *WRKY* genes was also enhanced by the *F. solani* infection. Following the inoculation with *F. solani*, the relative expression values of 5 *WRKY* genes (*PnWRKY5*/*11*/*15*/*25*/*28*) were maximum at 48 hpi (Figure 2). Notably, the *PnWRKY6*/*21*/*22* expression levels were highest during the early stage of *F. solani* infection. The expression levels of *PnWRKY9* and *PnWRKY30* were maximum at 72 hpi. These *WRKY* genes were clearly responsive to JA signaling and *F. solani* in *P. notoginseng* roots.

**Figure 3:**
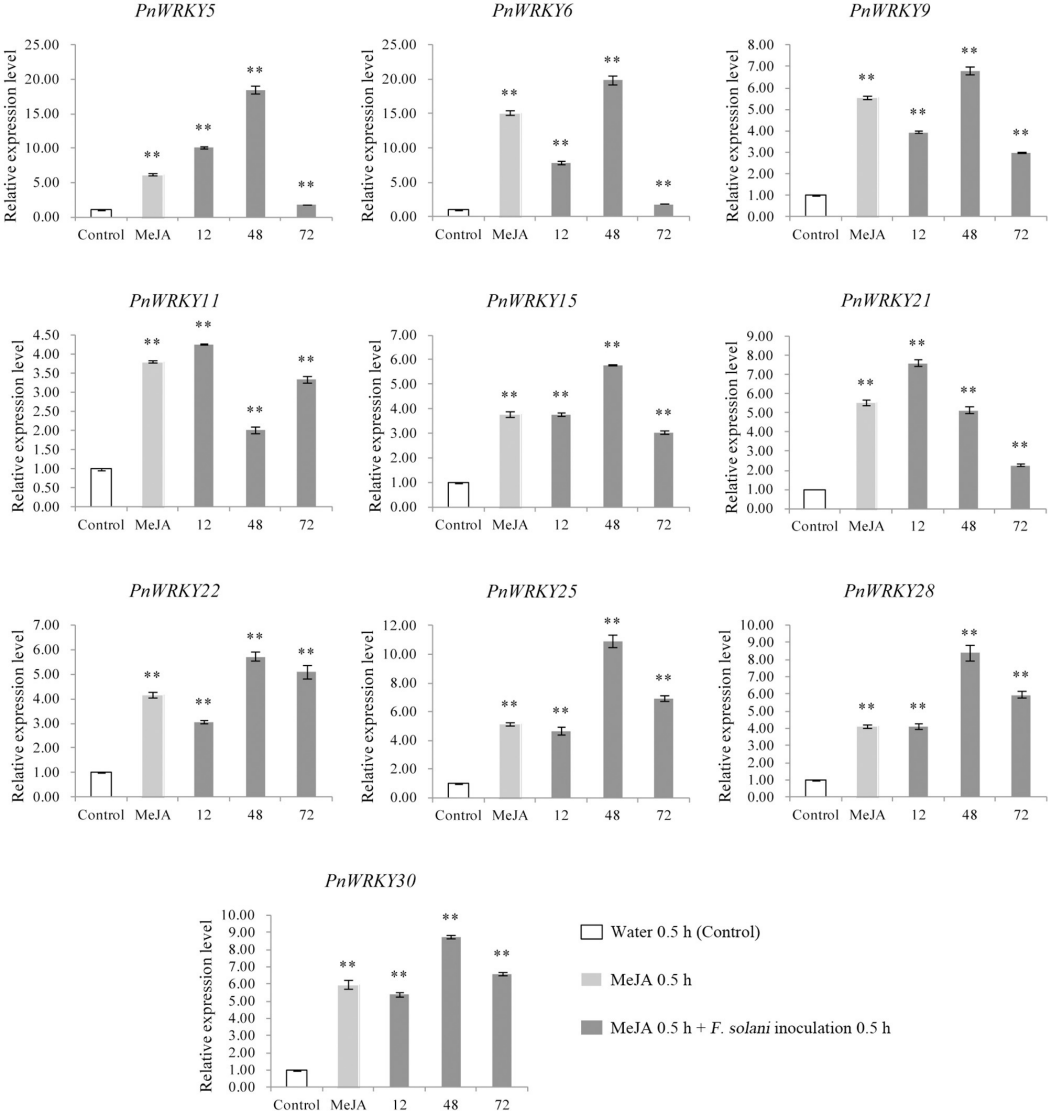
The expression levels of *PnWRKYs* were estimated in *P. notoginseng* roots pretreated with MeJA followed by *F. solani* infection. *Panax notoginseng* roots were pre-treated with MeJA, and after treatment for 24 h, the treated-roots were further inoculated with *F. solani*. The MeJA-treated roots were collected after treatment for 24 h, and the inoculated roots were collected at 12, 48, and 72 h post *F. solani* inoculation. *P. notoginseng* roots treated with sterile water for 30 min were used as control. The 2^−ΔΔCt^ method and *t* test (**p < 0.01) were used to calculate the relative gene expression levels and statistical difference.

Furthermore, the *P. notoginseng* roots were pre-treated with MeJA and then inoculated with *F. solani* in order to analyze the effect of JA signal on *PnWRKYs* expression during *F. solani* infection. The result confirmed that these *WRKY* genes responded to MeJA pre-treatment, in addition, their transcription levels were further enhanced during *F. solani* infection (Figure 3). Following the inoculation with *F. solani*, the expression levels of eight *WRKY* genes (*PnWRKY5*/*6*/*9*/*15*/*22*/*25*/*28*/*30*) were maximum at 48 hpi (Figure 3). Notably, the *PnWRKY11* and *PnWRKY21* expression levels were highest at 12 hpi. Among the examined *PnWRKY* genes, the expression of *PnWRKY5* and *PnWRKY6* was induced the most by *F. solani* infection. The above data showed that *P. notoginseng WRKY* genes may cooperate with JA signal in response to *F. solani* infection.

### The Group IIc Member PnWRKY9 Was Localized in the Nucleus

**Figure 4:**
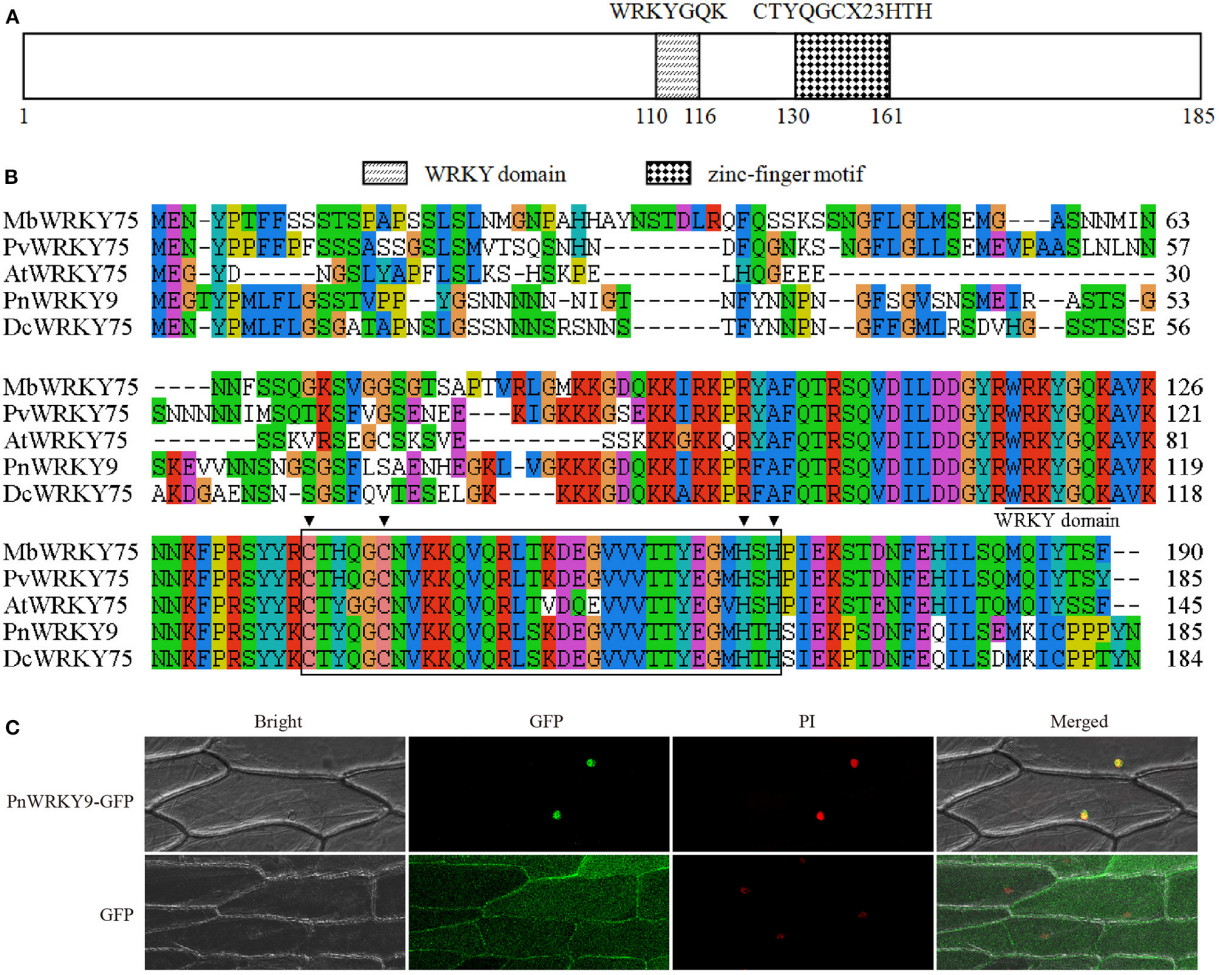
Sequence and subcellular localization analyses of PnWRKY9. **(A)** The structure schematic of PnWRKY9 protein. **(B)** Multiple sequence alignment of PnWRKY9 and homologous WRKY proteins using clustalx1.83 software. **(C)** Subcellular localization analysis of PnWRKY9-GFP fusion protein. Bright, white light field; GFP, green fluorescent field; PI, propidium iodide, which was used to indicate the nucleus; Merged, mixed field.

Considering the variability in the *P. notoginseng WRKY* gene expression patterns following the MeJA treatment and root rot pathogen infection, *PnWRKY9* was randomly selected for a functional analysis. An examination of protein homology indicated that PnWRKY9 was 74.59, 68, and 67.35% homologous to WRKY75 in carrot (*Daucus carota*, XP_017248451.1), pistachio (*Pistacia vera*, XP_031272738.1), and apple (*Malus baccata*, AhJ78583.1), respectively. Additionally, the alignment of PnWRKY9 with DcWRKY75, PvWRKY75, and MbWRKY75 revealed WRKYGQK and the CTYQGCX_23_HTH zinc finger motif were highly conserved (Figures 4A,B).

The PnWRKY9-GFP fusion protein was transiently expressed in onion epidermal cells. The green fluorescence of PnWRKY9-GFP was detected exclusively in the nucleus, whereas the green fluorescence of GFP alone was observed throughout the onion epidermal cells (Figure 4C). Moreover, the red fluorescence from the nuclear dye PI confirmed that PnWRKY9 is a nuclear protein.

### The Overexpression of *PnWRKY9* in Tobacco Conferred Resistance to *F. solani*

**Figure 5:**
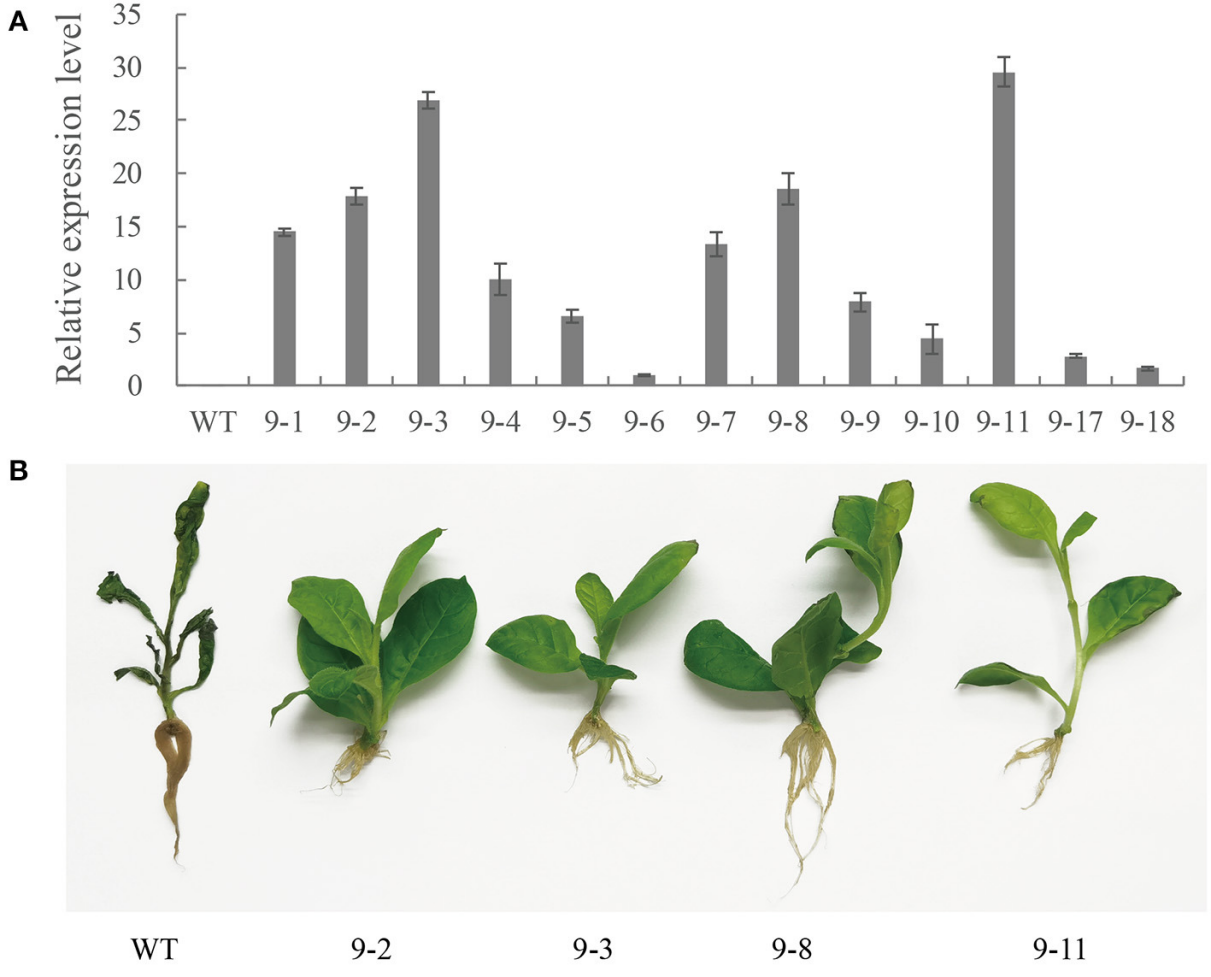
Analysis of *PnWRKY9*-OE tobacco. **(A)** *PnWRKY9* expression levels in the overexpression tobacco. The *PnWRKY9* was stably expressed in overexpression tobacco lines 9-1/2/3/4/5/6/7/8/9/10/11/17/18. **(B)** Resistance assay of *PnWRKY9*-OE tobacco against *F. solani*. The *PnWRKY9*-OE tobacco lines (9-2/3/8/11) and wild type (WT) tobacco roots were inoculated with *F. solani*. and the *PnWRKY9*-OE tobacco showed higher level of resistance to *F. solani* than the WT.

The qRT-PCR analysis revealed *PnWRKY9* expression levels varied among 13 T_2_ generation transgenic tobacco lines. The expression level was highest in lines 9-3 and 9-11. It was also relatively high in lines 9-1, 9-2, 9-4, 9-7, and 9-8 (Figure 5A). The data confirmed that *PnWRKY9* was stably expressed in the T_2_ transgenic tobacco lines. The tobacco roots were inoculated with *F. solani* and examined 7 days later. The *PnWRKY9*-OE tobacco lines had no obvious disease symptoms (i.e., plants were green and healthy). In contrast, the WT tobacco plants had withered leaves and roots that were black and rotted (Figure 5B). Thus, the overexpression of *PnWRKY9* clearly increased the resistance of transgenic tobacco plants to *F. solani*.

### The Transient Silencing of *PnWRKY9* by RNAi in *P. notoginseng* Leaves Increased the Susceptibility to *F. solani*

**Figure 6:**
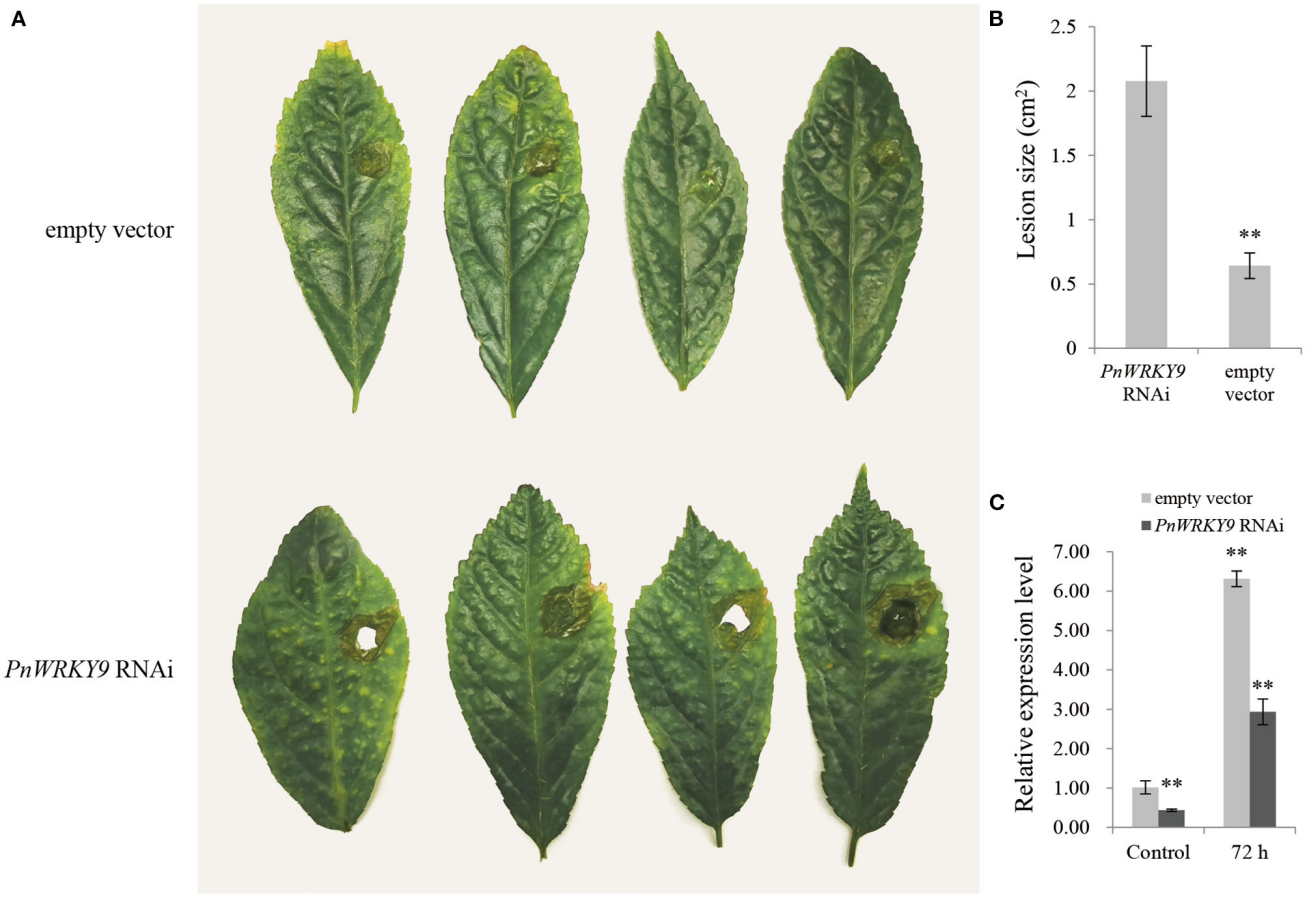
The transiently expression analysis of *PnWRKY9* RNAi vector in the leaves of *P. notoginseng*. **(A)** The symptoms of *P. notoginseng* leaves inoculated with *F. solani*, that were first infected with *A. tumefaciens* containing *PnWRKY9* RNAi vector. The *P. notoginseng* leaves with expressed empty RNAi vector and inoculated *F. solani* was as control. **(B)** The lesion size in *P. notoginseng* leaves expressing the *PnWRKY9* RNAi vector and empty RNAi vector, respectively (**p < 0.01). **(C)** The *PnWRKY9* expression levels in *P. notoginseng* leaves expressing *PnWRKY9* RNAi vector and empty RNAi vector, respectively. The results were calculated by the 2^−ΔΔCt^ method and analyzed by the *t* test (**p < 0.01).

The *PnWRKY9* RNAi fragments were transiently expressed in young *P. notoginseng* leaves. The leaves transformed with the RNAi empty vector served as the control samples. Although the extent of the damage caused by the *F. solani* infection varied, the lesions were almost 4-times larger on the leaves with the *PnWRKY9* RNAi fragments than on the control leaves (Figures 6A,B). Additionally, the qRT-PCR data indicated the *PnWRKY9* expression level in the leaves with the *PnWRKY9* RNAi fragments decreased to about half of that in the control leaves (Figure 6C). Accordingly, the decrease in *PnWRKY9* expression increased the susceptibility of *P. notoginseng* to *F. solani*.

### Several Plant Disease Resistance-Related Pathways, Including the JA Signaling Pathway, Were Significantly Activated in *PnWRKY9*-OE Tobacco

**Figure 7:**
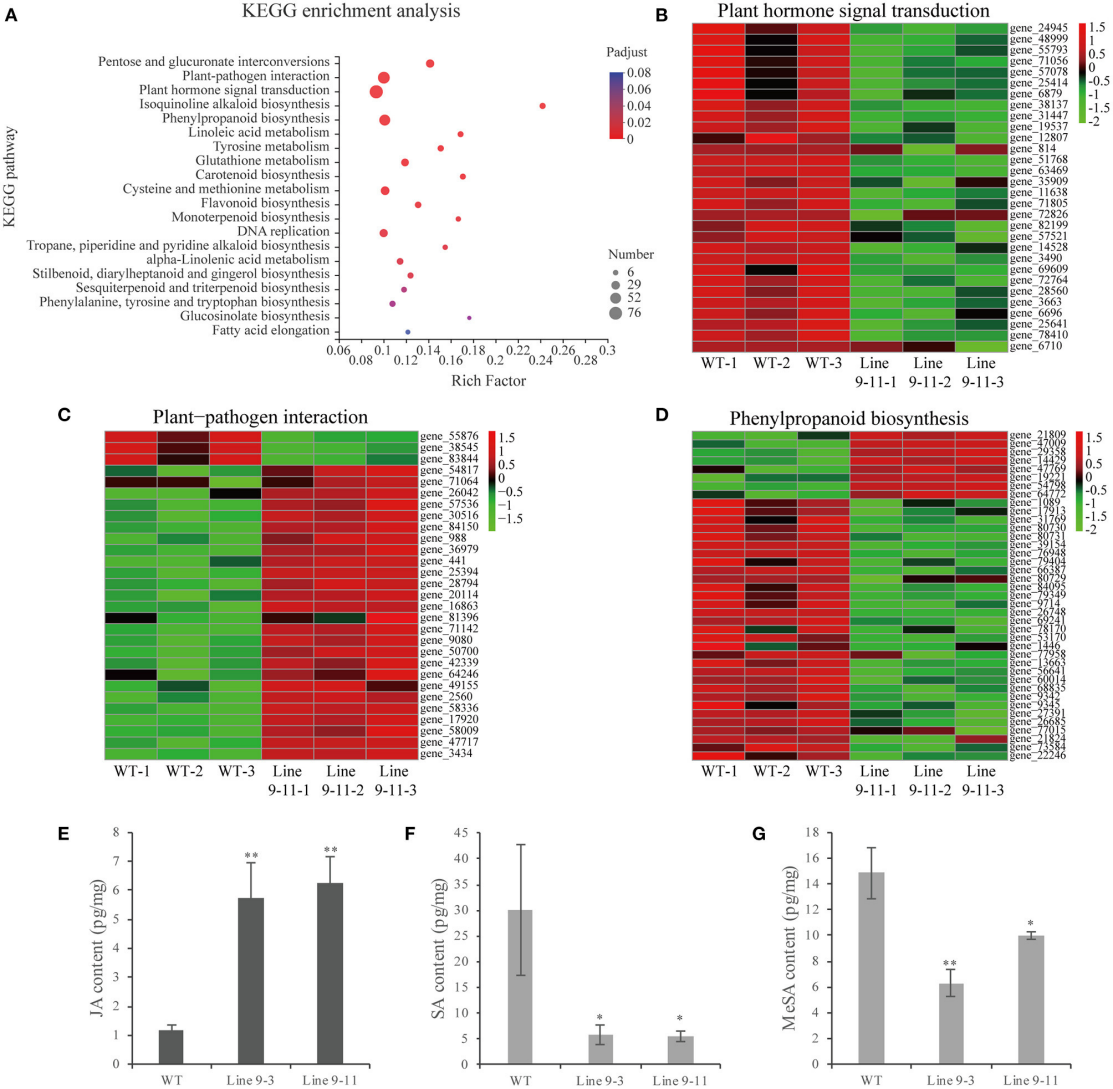
RNA sequencing analysis and JA/SA content in *PnWRKY9*-OE tobacco. **(A)** KEGG enrichment analysis of DEGs in *PnWRKY9*-OE tobacco line 9-11 compared with WT tobacco. The color showed the significance level, and the bubble size indicated the numbers of the enriched genes. **(B–D)** DEGs heat map in plant hormone signal, plant-pathogen interactions, and phenylpropanoid biosynthesis pathways. The red and green indicated the up-regulated and down-regulated gene expression, respectively. **(E–G)** JA, SA, and MeSA contents in *PnWRKY9*-OE tobacco line 9-3/11 and the WT tobacco (*p < 0.05; **p < 0.01).

The RNA-seq analysis of *PnWRKY9*-OE tobacco and WT tobacco detected 4,286 DEGs, including 976 up-regulated genes and 3,310 down-regulated genes. The KEGG analysis of the DEGs revealed that the three most significantly enriched pathways were plant hormone signal transduction, plant–pathogen interaction, and phenylpropanoid biosynthesis (Figure 7A). There were 76 DEGs associated with the plant hormone signal transduction pathway (Figure 7B). Some related genes of JA biosynthesis and signal transduction pathway were significantly up-regulated (Supplementary Table 5), such as *allene oxide synthase* and *bZIP transcription factor*, moreover, the expression of some JA signal repressor genes was down-regulated including *JAZs* and *MYC2*. However, the expression of SA biosynthesis related genes including *pyridoxal phosphate-dependent transferases, amidase, aspartate aminotransferase 3*, and *tyrosine transaminase* were down-regulated. The 61 DEGs involved in the plant–pathogen interaction pathway (Figure 7C) included up-regulated genes encoding heat shock protein 90 (HSP90), which is associated with plant hypersensitive responses (HRs), many disease resistance-related proteins, and cytochrome P450. In contrast, the expression of *WRKY33* encoding an HR-suppressor was down-regulated. Moreover, 52 DEGs were associated with the phenylpropanoid biosynthesis pathway, including some up-regulated genes encoding the peroxidase superfamily protein, UDP-glucosyl transferases, and cinnamoyl CoA reductase (Figure 7D).

The RNA-seq analysis indicated that the JA signaling pathway was activated, but the SA signaling pathway was suppressed, in *PnWRKY9*-OE tobacco. Therefore, the accumulation of endogenous JA and SA in *PnWRKY9*-OE tobacco and WT tobacco was analyzed to validate the RNA-seq data. The JA content was higher in *PnWRKY9*-OE tobacco than in WT tobacco (Figure 7E), whereas the opposite trend was observed for the SA and methyl salicylate contents (Figures 7F,G). These observations were consistent with the RNA-seq data. Accordingly, PnWRKY9 and the JA signaling pathway may cooperatively enhance disease resistance.

### The PnWRKY9 Recombinant Protein Can Bind Specifically to the W-Box in the Promoter of the *F. solani* Resistance-Related Gene *PnDEFL1*

**Figure 8:**
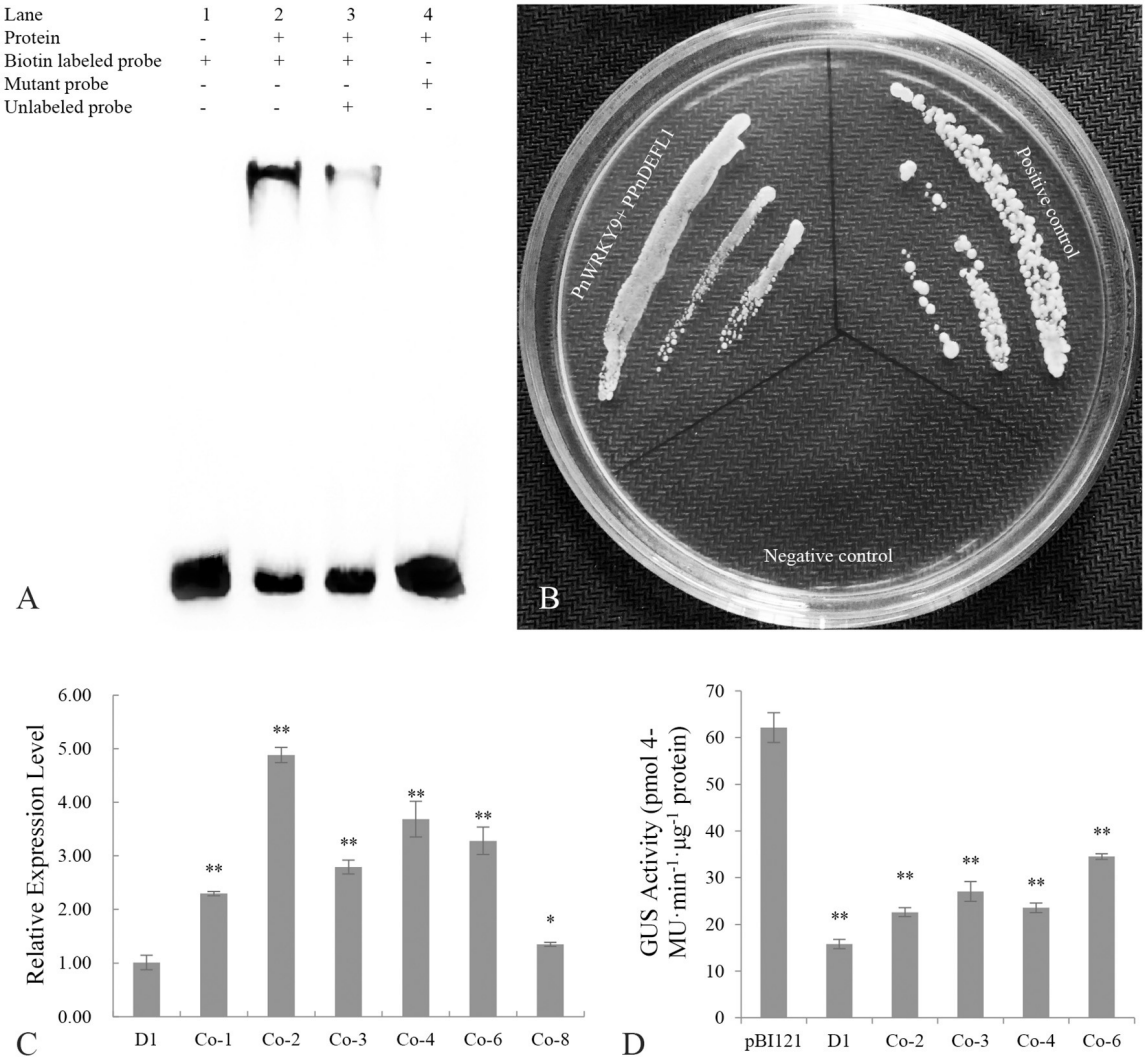
Transcriptional regulation of PnWRKY9 on PPnDEFL1. **(A)** EMSA assay verified the specific binding of PnWRKY9 with PPnDEFL1. Lane 1, the biotin labeled free probes containing the W-box sequence of PPnDEFL1; Lane 2, PnWRKY9 can interact with biotin labeled probes containing W-box sequence of PPnDEFL1; Lane 3, PnWRKY9 showed the interaction with the unlabeled probes and biotin labeled probes containing W-box sequence of PPnDEFL1; Lane 4, PnWRKY9 cannot interact with the mutant probes labeled by biotin. **(B)** Y1H assay verified the transcriptional activation of PnWRKY9 on PPnDEFL1. PnWRKY9 + PPnDEFL1, pGADT7-*PnWRKY9* and PPnDEFL1-pAbAi co-transformed yeast cells; Positive control, p53-pAbAi and pGADT7-*p53* co-transformed yeast cells; Negative control, PPnDEFL1-pAbAi and pGADT7-*P53* co-transformed yeast cells. **(C)** Analysis of *GUS* gene expression level in PPnDEFL1-*GUS*/*PnWRKY9* co-expression tobacco (Co-1/2/3/4/6/8). The transgenic tobacco (D1) just expressing the PPnDEFL1-*GUS* was control. **(D)** Determination of GUS activity in PPnDEFL1-*GUS*/*PnWRKY9* co-expression tobacco (Co-2/3/4/6). The transgenic tobacco just expressing the PPnDEFL1-*GUS* (D1) and pBI121-*GUS* were control (*p < 0.05; **p < 0.01).

The promoter sequence of a JA-responsive and *F. solani* resistance-related gene (*PnDEFL1*) was cloned for an analysis of the interaction between PnWRKY9 and a *P. notoginseng* disease resistance-related gene. The PPnDEFL1 sequence (475 bp) was obtained by genome walking. The promoter sequence was predicted to contain many *cis-*elements (Supplementary Table 3), including G-box, MYB, and W-box. Moreover, the PnWRKY9 recombinant protein was expressed in *E. coli* cells and purified to verify the specific binding between PnWRKY9 and PPnDEFL1. The EMSA results revealed a lack of bands shifts in lanes 1 and 4 (Figure 8A), which, respectively, corresponded to the free probes (W-box sequence of PPnDEFL1) without PnWRKY9 and the mutant probes (mutated W-box sequence) with PnWRKY9. Therefore, PnWRKY9 did not bind to the mutant W-box sequence. Specifically, bands shifts were detected in lanes 2 and 3 (Figure 8A). Moreover, the band shift in lane 2 was more obvious than in lane 3, likely because the abundance of competitor probes without biotin was 50-times greater than the abundance of free probes. These results indicated that PnWRKY9 can bind specifically to the W-box sequence of PPnDEFL1.

### PnWRKY9 Transcriptionally Activated *PnDEFL1* in Yeast and Tobacco

The ability of PnWRKY9 to activate the transcription of *PnDEFL1* was assessed in a Y1H assay (Figure 8B). The Y1Hgold yeast cells with p53-pAbAi and pGADT7-*P53* were used as the positive control, whereas the Y1Hgold yeast cells with PPnDEFL1-pAbAi and pGADT7-*P53* served as the negative control. The Y1Hgold yeast cells with PPnDEFL1-pAbAi/pGADT7-*PnWRKY9* grew normally on the SD/–Leu medium supplemented with 200 ng/mL AbA (i.e., similar to the control). In contrast, the negative control cells were unable to survive on the selective medium. The fusion protein GAL4-PnWRKY9 in the yeast cells transformed with the prey vector pGADT7-*PnWRKY9* was expected to bind to the *cis*-element W-box in PPnDEFL1 and activate the expression of *AUR1-C* in the pAbAi recombinant plasmid, enabling cells to grow normally on the SD/–Leu/AbA medium. The Y1H results confirmed PnWRKY9 can activate *PnDEFL1* transcription.

The *GUS* under the control of PPnDEFL1 was inserted into WT tobacco and *PnWRKY9*-OE tobacco. The subsequent qRT-PCR analysis revealed that *GUS* expression differed between the PPnDEFL1-*GUS*/*PnWRKY9*-OE and PPnDEFL1-*GUS* transgenic tobacco plants. The highest expression level, which was detected in line Co-2, was 3.88-times higher than that in the control (i.e., transgenic tobacco with only PPnDEFL1-*GUS*, D1). The second highest expression level was detected in line Co-4. The expression levels in Co-1, Co-3, Co-6, and Co-8 were, respectively, 1.3-, 1.8-, 2.3-, and 0.4-times higher than the control expression level (Figure 8C). Hence, lines Co-2, Co-3, Co-4, and Co-6 (PPnDEFL1-*GUS*/*PnWRKY9*-OE tobacco) were selected for an examination of GUS activity. Compared with the D1 control, the GUS activity was significantly higher in lines Co-2, Co-3, Co-4, and Co-6 (Figure 8D). Specifically, the GUS activity of the PPnDEFL1-*GUS*/*PnWRKY9*-OE tobacco exceeded 20 pmol 4-MU min^−1^ μg^−1^ protein, whereas the GUS activity of the control was 15 pmol 4-MU min^−1^ μg^−1^ protein. These observations suggested that PPnDEFL1 was activated by PnWRKY9.

## Discussion

The recent development of high-throughput sequencing technology has resulted in the identification of an increasing number of *WRKY* genes in various plant species. For example, an RNA-seq analysis identified 58 *WRKY* genes in eggplant (*Solanum melongena*), among which *SmWRKY26* and *SmWRKY32* were revealed to negatively regulate the cold stress tolerance of eggplant on the basis of virus-induced gene silencing (VIGS) (Yang et al., 2020). The *Arabidopsis thaliana WRKY* gene family comprises 74 genes, including *AtWRKY38* and *AtWRKY62*, which are involved in defense responses to *Pseudomonas aeruginosa*, and *AtWRKY57*, which helps regulate senescence (Birkenbihl et al., 2018). The WRKY domain and the zinc finger motif are conserved structures that are used to divide WRKY TFs into different groups (Rushton et al., 2010). In this study, 30 *P. notoginseng WRKY* genes were identified by analyzing transcriptome sequencing data (Liu D. et al., 2019). The conserved domains and phylogenetic relationships among *P. notoginseng* WRKY TFs were examined. Additionally, the 30 WRKY TFs in *P. notoginseng* were divided into three groups, which is in accordance with the classification of WRKYs in other plants including pepper (*C. annuum*) and eggplant (Zheng et al., 2019; Yang et al., 2020). As regards the *WRKY* gene evolution, the ancient group IIc *WRKY* genes were considered to be ancestor of all *WRKY* genes, therefore, group IIc WRKYs in plant were the most diverse members (Luise et al., 2013). In the phylogenetic tree of PnWRKYs, some coefficients located in left branch of group IIc were big, but those in the right branch were small, which was similar to the phylogenetic tree of wild rice (*Oryza nivara*) group IIc WRKYs (Xu et al., 2016). These results hinted that the group IIc members of WRKY super gene family had rich diversity. Moreover, because the *P. notoginseng* genome has been sequenced (Chen W. et al., 2017), the *WRKY* genes isolated in this study provide the basis for future investigations conducted to identify all members of the *P. notoginseng WRKY* superfamily.

Earlier research demonstrated that TFs involved in various plant physiological processes are usually responsive to treatment with multiple plant hormones (Jiang J. et al., 2017). The expression of *TaWRKY40-D*, which encodes a positive regulator of leaf senescence in wheat (*Triticum aestivum*), is reportedly up-regulated after JA and ABA treatments (Zhao L. et al., 2020). In lotus (*N. nucifera*) roots, *NnWRKY40a* and *NnWRKY40b* expression is significantly induced following a JA treatment (Li et al., 2019). Similarly, the expression of *GbWRKY20* in *Ginkgo biloba* is up-regulated at 12 h after the application of exogenous MeJA (Zhou et al., 2021). In another study, SA and JA treatments enhanced the expression of *PacMYBA* in sweet cherry (*Prunus avium* L.) leaves; the heterologous expression of this gene in *A. thaliana* increases the resistance to *Pseudomonas syringae* pv. *tomato* DC3000 (Shen et al., 2017). In the current study, the expression levels of several *WRKY* genes in *P. notoginseng* roots were up-regulated to varying degrees by the MeJA treatment. Moreover, the expression levels of 10 *PnWRKY* genes increased further in response to an *F. solani* infection. Thus, these *P. notoginseng* WRKY TFs and the JA signaling pathway may be involved in the early defense response to *F. solani*.

Proteins in plant cells are generally functional only in specific subcellular locations (Yousefi et al., 2021). Hence, analyzing the subcellular localization of proteins may provide important clues regarding protein functions. Most WRKY TFs are nuclear proteins (Zhao Y. et al., 2019). For example, in sugarcane (*Saccharum* spp.), WRKY3 is localized in the nucleus (Wang et al., 2018). A laser confocal microscopy-based examination of *Populus tremula* confirmed WRKY18 and WRKY35 are localized in the nucleus (Jiang Y. et al., 2017). Similarly, *P. notoginseng* WRKY9 was identified as a nuclear protein in the present study. Furthermore, the overexpression of *PnWRKY9* in tobacco was observed to enhance the resistance to *F. solani*. In accordance with this finding, the transient expression of the *PnWRKY9* RNAi fragment in *P. notoginseng* leaves increased the susceptibility of the plants to an infection by *F. solani*. The transcriptional regulation of TF genes occurs in the nucleus. Therefore, the identification of PnWRKY9 as a nuclear protein and the results of the reverse genetics analysis in this study confirmed that PnWRKY9 is a positive regulator of the root rot defense response in *P. notoginseng*.

Regulatory genes are crucial for the transcriptome reprogramming during plant responses to pathogens, and their functions are further amplified by the crosstalk of some signal transduction pathways (Shen et al., 2017). The expression levels of *CsWRKY50* and some hormone signaling pathway-related genes, including JA-responsive genes and genes involved in SA biosynthesis, are up-regulated in cucumber (*Cucumis sativus*) during a *Pseudoperonospora cubensis* infection (Luan et al., 2019). The overexpression of cotton (*Gossypium barbadense*) *WRKY1* enhances the resistance to *Verticillium dahliae*, but the silencing of this gene by VIGS has suppressive effects on JA- and ET-induced defense activities (Zhang et al., 2019). The cross-talk of hormone signal transduction pathways is complex, for example, the SA and JA signal pathways showed synergistic or antagonistic effects under different conditions. *Arabidopsis* WRKY70 is a positive regulator of SA-signaling pathway in response to *P. syringae*, but it functions as a negative regulator of JA-inducible genes (Li et al., 2004). In addition, the decreased expression of cotton (*G. hirsutum*) *WRKY70* enhanced the resistance to *V. dahliae*, which resulted in up-regulated expression of JA responding genes and down-regulated expression of SA signaling pathway genes (Xiong et al., 2020). In the present study, the comparison between *PnWRKY9-*overexpressing tobacco and the control plants revealed plant hormone signal transduction and plant–pathogen interaction were the most enriched KEGG pathways among the DEGs. Some JA signaling pathway genes were up-regulated, nevertheless some SA signaling pathway genes were down-regulated. Additionally, the JA content was higher in *PnWRKY9*-OE tobacco than in WT tobacco, whereas the opposite trend was detected for the SA level. These findings imply that PnWRKY9 may synergistically JA signal and inhibit SA accumulation to regulate *P. notoginseng* defenses against the root rot pathogen.

The WRKY TFs bind to the W-box sequence in the promoters of their target genes, including disease resistance-related genes, through the WRKY domain, and then activate or repress the expression of the target genes (Li et al., 2021a). The interactions between WRKY TFs and their target genes have been elucidated in a variety of plant species on the basis of EMSA and Y1H analyses. In apple (*Malus* × *domestica*) WRKY46 binds to the *MdPBS3.1* promoter, which includes a W-box sequence, and the resulting activation of *MdPBS3.1* expression enhances the resistance to powdery mildew (Zhao et al., 2019b). In *Citrus sinensis*, WRKY65 regulates the expression of some *Penicillium digitatum* resistance-related genes (e.g., *CsRbohB, CsRbohD, CsCDPK33*, and *CsPR10*) by binding to the W-box in their promoters (Wang et al., 2021). In this study, EMSA and Y1H results indicated that PnWRKY9 directly regulates the expression of the *F. solani* resistance-related defensin gene *PnDEFL1*. This was confirmed by the significantly increased *GUS* expression level and GUS activity in the PPnDEFL1-*GUS/PnWRKY9*-OE tobacco. Thus, the regulation of *PnDEFL1* expression may partially explain the synergism between JA and PnWRKY9 during the molecular interaction between *P. notoginseng* and the root rot pathogen.

**Figure 9:**
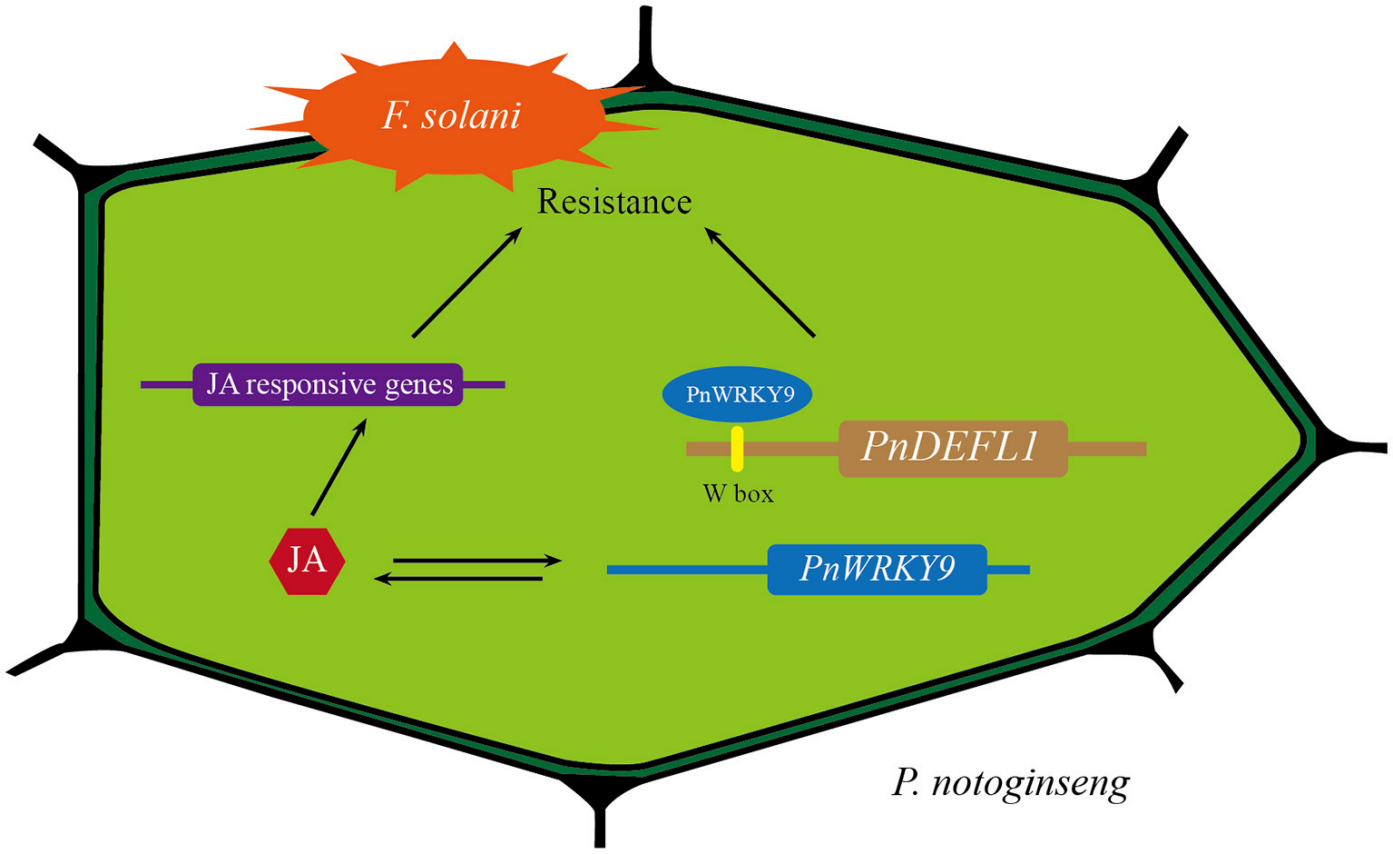
A model of positive regulation of PnWRKY9 in *P. notoginseng* in response to *F. solani* infection. After *P. notoginseng* was infected by root rot pathogen *F. solani*, PnWRKY9 activated the expression of JA-responsive resistance gene *PnDEFL1* by specifically binding W-box in *PnDEFL1* promoter, which enhanced resistance to *F. solani*. Moreover, the activated JA signaling induced the expression of JA-responsive genes to boost defense response against *F. solani*.

In summary, we proposed a model of positive regulation of PnWRKY9 in *P. notoginseng* during response to *F. solani* infection (Figure 9). When *P. notoginseng* plants were threatened by root rot pathogen *F. solani, PnWRKY9* was greatly induced to be expressed, and then PnWRKY9 specifically bound to the W-box sequence in promoter of *P. notoginseng* disease resistance gene *PnDEFL1*, which activated the expression of *PnDEFL1* and enhanced the resistance of *P. notoginseng* to *F. solani*. Meanwhile, the accumulation of JA in *P. notoginseng* was also induced by *F. solani*, and the expression of JA-responsive resistance genes in *P. notoginseng*, such as *PnSN1* and *PnDEFL* were further induced by the JA signaling to enhance resistance to root rot.

## Conclusion

In this study, we identified *P. notoginseng WRKY* genes and determined that the expression of 10 *PnWRKY* genes was inducible by the MeJA treatment and *F. solani* infection. Moreover, *PnWRKY9* was revealed to encode a nuclear protein that positively regulates the resistance of *P. notoginseng* to *F. solani*. Additionally, PnWRKY9 and the JA signaling pathway synergistically affect the defense response of *P. notoginseng* to root rot through the regulated expression of the defensin gene *PnDEFL1*.

## Data Availability Statement

The original contributions presented in the study are publicly available. The data can be found in the NCBI: PnWRKY1-PnWRKY30 (accession numbers KM925138, OM273030-OM273058); RNA sequencing data of PnWRKY9 transgenic tobacco accession number PRJNA827632. The sequence of PnDEFL1 promoter was showed in the Supplementary Materials.

## Author Contributions

LZ: methodology, data curation, experiment execution, and writing—original draft preparation. BQ: methodology, material collection, and experiment execution. LS: validation. HW: software and conceptualization. XC and FG: supervision. DL: methodology, supervision, and writing—reviewing and editing. All authors contributed to the article and approved the submitted version.

## Funding

This work was financially supported by one grant received from the National Natural Sciences Foundation of China (Grant No. 82060693).

## Conflict of Interest

The authors declare that the research was conducted in the absence of any commercial or financial relationships that could be construed as a potential conflict of interest.

## Publisher's Note

All claims expressed in this article are solely those of the authors and do not necessarily represent those of their affiliated organizations, or those of the publisher, the editors and the reviewers. Any product that may be evaluated in this article, or claim that may be made by its manufacturer, is not guaranteed or endorsed by the publisher.

## References

[B1] Afrin S. Huang J. J. Luo Z. Y. (2015). JA-mediated transcriptional regulation of secondary metabolism in medicinal plants. Sci. Bull. 60, 1062–1072. 10.1007/s11434-015-0813-0

[B2] Allen G. C. Flores-Vergara M. A. Krasynanski S. Kumar S. Thompson W. F. (2006). A modified protocol for rapid DNA isolation from plant tissues using cetyltrimethylammonium bromide. Nat. Protoc. 1, 2320–2325. 10.1038/nprot.2006.384 17406474

[B3] Bawa G. Feng L. Yan L. Du Y. Shang J. Sun X.. (2019). Pre-treatment of salicylic acid enhances resistance of soybean seedlings to *Fusarium solani*. Plant Mol. Biol. 101, 315–323. 10.1007/s11103-019-00906-x 31392474

[B4] Birkenbihl R. P. Kracher B. Ross A. Kramer K. Finkemeier I. Somssich I. E. (2018). Principles and characteristics of the Arabidopsis WRKY regulatory network during early MAMP-triggered immunity. Plant J. 96, 487–502. 10.1111/tpj.14043 30044528

[B5] Caroline F. A. Olubukola O. (2013). Integrated management strategies for tomato *Fusarium wilt*. Biocontrol Sci. 18, 117–127. 10.4265/bio.18.117 24077535

[B6] Catinot J. Huang J. B. Huang P. Y. Tseng M. Y. Chen Y. L. Gu S. Y.. (2015). ETHYLENE RESPONSE FACTOR 96 positively regulates *Arabidopsis* resistance to necrotrophic pathogens by direct binding to GCC elements of jasmonate - and ethylene-responsive defence genes. Plant Cell Environ. 38, 2721–2734. 10.1111/pce.12583 26038230

[B7] Chen L. Zhang L. Xiang S. Chen Y. Zhang H. Yu D. (2021). The transcription factor WRKY75 positively regulates jasmonate-mediated plant defense to necrotrophic fungal pathogens. J. Exp. Bot. 72, 1473–1489. 10.1093/jxb/eraa529 33165597 PMC7904156

[B8] Chen R. He H. Yang Y. Qu Y. Ge F. Liu D. (2017). Functional characterization of a pathogenesis-related protein family 10 gene, *LrPR10-5*, from *Lilium regale* Wilson. Australas. Plant Pathol. 46, 251–259. 10.1007/s13313-017-0485-0

[B9] Chen W. Kui L. Zhang G. Zhu S. Zhang J. Wang X.. (2017). Whole-genome sequencing and analysis of the Chinese herbal plant *Panax notoginseng*. Mol. Plant. 10, 899–902. 10.1016/j.molp.2017.02.010 28315474

[B10] Hao W. Gray M. A. Forster H. Adaskaveg J. E. (2019). Evaluation of new oomycota fungicides for management of phytophthora root rot of citrus in California. Plant Dis. 103, 619–628. 10.1094/PDIS-07-18-1152-RE 30789317

[B11] Holsters M. de Waele D. Depicker A. Messens E. van Montagu M. Schell J. (1978). Transfection and transformation of *Agrobacterium tumefaciens*. Mol. Gen. Genet. 163, 181–187. 10.1007/BF00267408 355847

[B12] Horsch R. Rogers S. Fraley R. (1985). Transgenic plant. Cold Spring Harb. Symp. Quant. Biol. 50, 433–437. 10.1101/SQB.1985.050.01.054 3868487

[B13] Hussain A. Li X. Weng Y. Liu Z. Ashraf M. F. Noman A.. (2018). CaWRKY22 acts as a positive regulator in pepper response to *Ralstonia solanacearum* by constituting networks with CaWRKY6, CaWRKY27, CaWRKY40, and CaWRKY58. Int. J. Mol. Sci. 19:1426. 10.3390/ijms19051426 29747470 PMC5983767

[B14] Ifnan Khan M. Zhang Y. Liu Z. Hu J. Liu C. Yang S.. (2018). *CaWRKY40b* in pepper acts as a negative regulator in response to *Ralstonia solanacearum* by directly modulating defense genes including *CaWRKY40*. Int. J. Mol. Sci. 19:1403. 10.3390/ijms19051403 29738468 PMC5983674

[B15] Jiang J. Ma S. Ye N. Jiang M. Cao J. Zhang J. (2017). WRKY transcription factors in plant responses to stresses. J. Integr. Plant Biol. 59, 86–101. 10.1111/jipb.12513 27995748

[B16] Jiang Y. Guo L. Ma X. Zhao X. Jiao B. Li C.. (2017). The WRKY transcription factors PtrWRKY18 and PtrWRKY35 promote *Melampsora* resistance in *Populus*. Tree Physiol. 37, 665–675. 10.1093/treephys/tpx008 28338710

[B17] Jiao Z. Sun J. Wang C. Dong Y. Xiao S. Gao X.. (2018). Genome-wide characterization, evolutionary analysis of WRKY genes in Cucurbitaceae species and assessment of its roles in resisting to powdery mildew disease. PLoS ONE. 13:e0199851. 10.1371/journal.pone.0199851 30589839 PMC6307730

[B18] Khaliq A. Alam S. Khan I. U. Khan D. Naz S. Zhang Y.. (2020). Integrated control of dry root rot of chickpea caused by *Rhizoctonia bataticola* under the natural field condition. Biotechnol. Rep. 25:e00423. 10.1016/j.btre.2020.e00423 31993345 PMC6976924

[B19] Li J. Brader G. Palva E. T. (2004). The WRKY70 transcription factor: a node of convergence for jasmonate-mediated and salicylate-mediated signals in plant defense. Plant Cell. 16, 319–331. 10.1105/tpc.016980 14742872 PMC341906

[B20] Li J. Xiong Y. Li Y. Ye S. Yin Q. Gao S.. (2019). Comprehensive analysis and functional studies of WRKY transcription factors in *Nelumbo nucifera*. Int. J. Mol. Sci. 20:5006. 10.3390/ijms20205006 31658615 PMC6829473

[B21] Li S. Hai J. Wang Z. Deng J. Liang T. Su L.. (2021a). *Lilium regale* Wilson WRKY2 regulates chitinase gene expression during the response to the root rot pathogen *Fusarium oxysporum*. Front. Plant Sci. 12:741463. 10.3389/fpls.2021.741463 34646290 PMC8503523

[B22] Li S. Liu G. Pu L. Liu X. Wang Z. Zhao Q.. (2021b). WRKY transcription factors actively respond to *Fusarium oxysporum* in *Lilium regale* Wilson. Phytopathology 111, 1625–1637. 10.1094/PHYTO-10-20-0480-R 33576690

[B23] Liu D. Zhao Q. Cui X. Chen R. Li X. Qiu B.. (2019). A transcriptome analysis uncovers *Panax notoginseng* resistance to *Fusarium solani* induced by methyl jasmonate. Genes Genomics 41, 1383–1396. 10.1007/s13258-019-00865-z 31493262

[B24] Liu X. Li D. Zhang S. Xu Y. Zhang Z. (2019). Genome-wide characterization of the rose (*Rosa chinensis*) WRKY family and role of RcWRKY41 in gray mold resistance. BMC Plant Biol. 19:522. 10.1186/s12870-019-2139-6 31775626 PMC6882016

[B25] Luan Q. Chen C. Liu M. Li Q. Wang L. Ren Z. (2019). *CsWRKY50* mediates defense responses to *Pseudoperonospora cubensis* infection in *Cucumis sativus*. Plant Sci. 279, 59–69. 10.1016/j.plantsci.2018.11.002 30709494

[B26] Luise H. B. Nina M. F. Klaus H. Oliver K. Dierk W. (2013). Elucidating the evolutionary conserved DNA-binding specificities of WRKY transcription factors by molecular dynamics and in vitro binding assays. Nucleic Acids Res. 41, 9764–9778. 10.1093/nar/gkt732 23975197 PMC3834811

[B27] Ma Y. N. Chen C. J. Li Q. Q. Xu F. R. Cheng Y. X. Dong X. (2019). Monitoring antifungal agents of *Artemisia annua* against *Fusarium oxysporum* and *Fusarium solani*, associated with *Panax notoginseng* root-rot disease. Molecules 24:213. 10.3390/molecules24010213 30626142 PMC6337599

[B28] Qiu B. Zhang Y. Wang Q. Wang Z. Chen H. Cui X.. (2020). *Panax notoginseng* snakin gene increases resistance to *Fusarium solani* in transgenic tobacco. Ind. Crops Prod. 157:112902. 10.1016/j.indcrop.2020.112902

[B29] Rushton P. J. Somssich I. E. Ringler P. Shen Q. J. (2010). WRKY transcription factors. Trends Plant Sci. 15, 247–258. 10.1016/j.tplants.2010.02.006 20304701

[B30] Schollenberger M. Muller H. M. Rufle M. Suchy S. Planck S. Drochner W. (2005). Survey of *Fusarium toxins* of plant origin marketed. Int. J. Food Microbiol. 97, 317–326. 10.1016/j.ijfoodmicro.2004.05.001 15582742

[B31] Shen X. Guo X. Guo X. Zhao D. Zhao W. Chen J.. (2017). PacMYBA, a sweet cherry R2R3-MYB transcription factor, is a positive regulator of salt stress tolerance and pathogen resistance. Plant Physiol. Biochem. 112, 302–311. 10.1016/j.plaphy.2017.01.015 28126679

[B32] Wang L. Liu F. Zhang X. Wang W. Sun T. Chen Y.. (2018). Expression characteristics and functional analysis of the *ScWRKY3* gene from sugarcane. Int. J. Mol. Sci. 19:4059. 10.3390/ijms19124059 30558233 PMC6321069

[B33] Wang Q. Qiu B. L. Li S. Zhang Y. P. Cui X. M. Ge F.. (2019). A methyl jasmonate induced defensin like protein from *Panax notoginseng* confers resistance against *Fusarium solani* in transgenic tobacco. Biol. Plant. 63, 797–807. 10.32615/bp.2019.123

[B34] Wang W. Li T. Chen Q. Deng B. Deng L. Zeng K. (2021). Transcription factor CsWRKY65 participates in the establishment of disease resistance of citrus fruits to *Penicillium digitatum*. J. Agric. Food Chem. 69, 5671–5682. 10.1021/acs.jafc.1c01411 33988021

[B35] Wang X. Li J. Guo X. Ma Y. Qiao Q. Guo J. (2019). PlWRKY13: A transcription factor involved in abiotic and biotic stress responses in *Paeonia lactiflora*. Int. J. Mol. Sci. 20:5953. 10.3390/ijms20235953 31779255 PMC6928655

[B36] Xie W. Meng X. Zhai Y. Zhou P. Ye T. Wang Z.. (2018). *Panax Notoginseng* saponins: a review of its mechanisms of antidepressant or anxiolytic effects and network analysis on phytochemistry and pharmacology. Molecules 23:940. 10.3390/molecules23040940 29673237 PMC6017639

[B37] Xiong X. P. Sun S. C. Zhang X. Y. Li Y. J. Liu F. Zhu Q. H.. (2020). GhWRKY70D13 regulates resistance to *Verticillium dahliae* in cotton through the ethylene and jasmonic acid signaling pathways. Front. Plant Sci. 11:69. 10.3389/fpls.2020.00069 32158454 PMC7052014

[B38] Xu H. Watanabe K. A. Zhang L. Shen Q. J. (2016). WRKY transcription factor genes in wild rice Oryza nivara. DNA Res. 23, 311–323. 10.1093/dnares/dsw025 27345721 PMC4991837

[B39] Xu Y. P. Xu H. Wang B. Su X. D. (2020). Crystal structures of N-terminal WRKY transcription factors and DNA complexes. Protein Cell. 11, 208–213. 10.1007/s13238-019-00670-0 31734872 PMC7026264

[B40] Yang Y. Liu J. Zhou X. Liu S. Zhuang Y. (2020). Identification of WRKY gene family and characterization of cold stress-responsive WRKY genes in eggplant. PeerJ. 8:e8777. 10.7717/peerj.8777 32211240 PMC7083166

[B41] Yousefi R. Jevdokimenko K. Kluever V. Pacheu-Grau D. Fornasiero E. F. (2021). Influence of subcellular localization and functional state on protein turnover. Cells 10:1747. 10.3390/cells10071747 34359917 PMC8306977

[B42] Zhang X. Liu J. Wu L. Wang Z. Zhang S. (2019). *GbWRKY1*, a member of the WRKY transcription factor family identified from *Gossypium barbadense*, is involved in resistance to *Verticillium wilt*. Biotechnol. Biotechnol. Equip. 33, 1354–1364. 10.1080/13102818.2019.1667873

[B43] Zhao L. Zhang W. Song Q. Xuan Y. Li K. Cheng L.. (2020). A WRKY transcription factor, TaWRKY40-D, promotes leaf senescence associated with jasmonic acid and abscisic acid pathways in wheat. Plant Biol. 22, 1072–1085. 10.1111/plb.13155 32609938

[B44] Zhao X. Y. Qi C. H. Jiang H. Zhong M. S. You C. X. Li Y. Y.. (2019a). MdHIR4 transcription and translation levels associated with disease in apple are regulated by MdWRKY31. Plant Mol. Biol. 101, 149–162. 10.1007/s11103-019-00898-8 31267255

[B45] Zhao X. Y. Qi C. H. Jiang H. Zhong M. S. Zhao Q. You C. X.. (2019b). MdWRKY46-enhanced apple resistance to *Botryosphaeria dothidea* by activating the expression of MdPBS3.1 in the salicylic acid signaling pathway. Mol. Plant Microbe Interact. 32, 1391–1401. 10.1094/MPMI-03-19-0089-R 31408392

[B46] Zhao Y. Yang Z. Ding Y. Liu L. Han X. Zhan J.. (2019). Over-expression of an R2R3 MYB Gene, *GhMYB73*, increases tolerance to salt stress in transgenic Arabidopsis. Plant Sci. 286, 28–36. 10.1016/j.plantsci.2019.05.021 31300139

[B47] Zheng J. Liu F. Zhu C. Li X. Dai X. Yang B.. (2019). Identification, expression, alternative splicing and functional analysis of pepper WRKY gene family in response to biotic and abiotic stresses. PLoS ONE. 14:e0219775. 10.1371/journal.pone.0219775 31329624 PMC6645504

[B48] Zhou M. L. Memelink J. (2016). Jasmonate-responsive transcription factors regulating plant secondary metabolism. Biotechnol. Adv. 34, 441–449. 10.1016/j.biotechadv.2016.02.004 26876016

[B49] Zhou T. Yang X. Wang G. Cao F. (2021). Molecular cloning and expression analysis of a WRKY transcription factor gene, *GbWRKY20*, from Ginkgo biloba. Plant Signal. Behav. 16:1930442. 10.1080/15592324.2021.1930442 34024256 PMC8331020

[B50] Zhu Z. An F. Feng Y. Li P. Xue L. A M.. (2011). Derepression of ethylene-stabilized transcription factors (EIN3/EIL1) mediates jasmonate and ethylene signaling synergy in *Arabidopsis*. Proc Natl Acad Sci USA. 108, 12539–12544. 10.1073/pnas.1103959108 21737749 PMC3145709

