# Supplementary material for: Panax notoginseng WRKY Transcription Factor 9 Is a Positive Regulator in Responding to Root Rot Pathogen Fusarium solani
Source: Front Plant Sci. 2022 Jul 14;13:930644. doi: 10.3389/fpls.2022.930644 (PMC9331302; doi:10.3389/fpls.2022.930644)
Supplement: Supplementary file 1 [file Data_Sheet_1.docx]

**Supplementary Tables:**

Table S1 The primers for qRT-PCR

| Target gene | Primer sequences |
| --- | --- |
| *PnWRKY5* | Forward: 5′GGGTATCCTTGCTGTTTGAGTAAT3′  Reverse: 5′TAGAACTCTGTGGCAACTCTCAACT3′ |
| *PnWRKY6* | Forward: 5′CGCATAGTTGTGTGTAGATGGAGTA3′  Reverse: 5′TAGTTGAGGGAGAGTTATCCATTTG3′ |
| *PnWRKY9* | Forward: 5′AATTGTCAGATGGCTTCTCTATGG3′  Reverse: 5′TTAATAACTCCAACGGTAGTGGAAG3′ |
| *PnWRKY11* | Forward: 5′AAGAAGCCCCTGATCTCTAAACA3′  Reverse: 5′CCCAGCTTCACTATCAACAAATACT3′ |
| *PnWRKY15* | Forward: 5′GTAGATTCAGAAAGCAGCCACCT3′  Reverse: 5′GCTGCTGTTGTAGTGGTAGAAGTTT3′ |
| *PnWRKY16* | Forward: 5′GCAACCCAACTTCAAAATCTACTAC3′  Reverse: 5′AATCTTGAGAGTTCAGTTGAGCAGA3′ |
| *PnWRKY21* | Forward: 5′CAAAGTGGGGTGGTTTCGCC3′  Reverse: 5′ATTCCTTGGATTTGGGCTGTT3′ |
| *PnWRKY22* | Forward: 5′AGTCGGATTCATCTCCTTCACC3′  Reverse: 5′CCATACTCACTCCAAACCTCCA3′ |
| *PnWRKY25* | Forward: 5′GTTGGGGTCCGCCGTAA3′  Reverse: 5′GCCGAGTGCCGACAGAATA3′ |
| *PnWRKY28* | Forward: 5′TCTTAACGGAGGGCGATACG3′  Reverse: 5′CCTACACCTCAGAACATAACCATCC3′ |
| *PnWRKY30* | Forward: 5′TTGGGGTGTCGCATCTAACTCT3′  Reverse: 5′TCCTTCAGTGCCTAATAAATCGG3′ |
| *PnACT2* | Forward: 5′TCCAAGGGTGAATATGATGAATCG3′  Reverse: 5′AACCTCTCCAAAGAGAATTTCTGAGT3′ |
| *GUS* | Forward: 5′CGGATACCCGTCCGCAAGT3′  Reverse: 5′GTGTGAGCGTCGCAGAACATT3′ |
| *NtACT* | Forward: 5′TCCCATTGAGCATGGAATAGTAAGC3′  Reverse: 5′TACATGGCAGGTACATTGAAAGTCT3′ |

Table S2 The primers used for *PnWRKY9* functional analysis

| Target genes | Primer sequences | Assays |
| --- | --- | --- |
| *PnWRKY9* | Forward: 5′GGATCCATGGAGGGTACTTATCCGATGC3′  Reverse: 5′TCTAGAATTATAGGGAGGAGGGCAAATCT3′ | Subcellular localization |
| *PnWRKY9* | Forward: 5′GAATTCATGGAGGGTACTTATCCGATGC3′  Reverse: 5′GGATCCTTAATTATAGGGAGGAGGGCAAATC3′ | Overexpression |
| *PnWRKY9* | Forward: 5′GGGGACAAGTTTGTACAAAAAAGCAGGCTGATGGAGGGTACTTATCCGATGC3′  Reverse: 5′GGGGACCACTTTGTACAAGAAAGCTGGGTCTTTAATTATAGGGAGGAGGGCAAATC3′ | RNAi |
| PPnDEFL1 | GSP1: 5′AGAGAGATTGTAATGAAGGTAGTTGGGCT3′  GSP2: 5′CGTTCTGGCATTGCTGTTCACAGTG3′ | Genome-walking |
| *PnWRKY9* | Forward: 5′GCCATGGCTGATATCGGATCCATGGAGGGTACTTATCCGATGCT3′  Reverse: 5′TTGTCGACGGAGCTCGAATTCCCATTATAGGGAGGAGGGCAAATCTT3′ | EMSA |
| Probe | GAGTCTAAGGAGATGGTTGGTCAATTGGGTGACCGATCGGAACTTGTTG | EMSA |
| Mutant probe | GAGTCTAAGGAGATGGTTGGATAATTGGGTGACCGATCGGAACTTGTTG | EMSA |
| PPnDEFL1 | Forward: 5′GGATCCAGATGTTCACCTTTTTAGGT3′  Reverse: 5′AAGCTTAAGTCCGTGTTTGATTTATA3′ | Y1H |
| *PnWRKY9* | Forward: 5′GAATTCATGGAGGGTACTTATCCGATGC3′  Reverse: 5′GGATCCTTAATTATAGGGAGGAGGGCAAATC3′ | Y1H |
| PPnDEFL1 | Forward: 5′AGTACTAGATGTTCACCTTTTTAGGT3′  Reverse: 5′TCTAGAAAGTCCGTGTTTGATTTATA3′ | Co-expression |

Table S3 The *cis*-elements in the PPnDEFL1

| The *cis*-element name | Sequence | Element function | Position(bp) |
| --- | --- | --- | --- |
| GATAMOTIF | GATAGGG | light responsive element | -25~31 |
| Box 4 | ATTAAT | a conserved DNA module involved in light responsiveness | -36~41 |
| MYB | TGGTTG | light responsive element | -188~193 |
| W-box | GGTCAA | ET、SA、MeJA responsive element | -193~198 |
| TGACGMOTIF | TGACG | MeJA responsive element | -304~308 |
| ATRICHELEMENT | ATATAAATCAA | ATBP-1 | -527~537 |

Table S4 KEGG enrichment information

| Num | Pathway id | Description | First Category | Second Category |
| --- | --- | --- | --- | --- |
| 76 | map04075 | Plant hormone signal transduction | Environmental Information Processing | Signal transduction |
| 61 | map04626 | Plant-pathogen interaction | Organismal Systems | Environmental adaptation |
| 52 | map00940 | Phenylpropanoid biosynthesis | Metabolism | Biosynthesis of other secondary metabolites |
| 32 | map00270 | Cysteine and methionine metabolism | Metabolism | Amino acid metabolism |
| 29 | map00040 | Pentose and glucuronate interconversions | Metabolism | Carbohydrate metabolism |
| 28 | map03030 | DNA replication | Genetic Information Processing | Replication and repair |
| 25 | map00480 | Glutathione metabolism | Metabolism | Metabolism of other amino acids |
| 18 | map00592 | alpha-Linolenic acid metabolism | Metabolism | Lipid metabolism |
| 17 | map00941 | Flavonoid biosynthesis | Metabolism | Biosynthesis of other secondary metabolites |
| 16 | map00350 | Tyrosine metabolism | Metabolism | Amino acid metabolism |
| 15 | map00950 | Isoquinoline alkaloid biosynthesis | Metabolism | Biosynthesis of other secondary metabolites |
| 15 | map00945 | Stilbenoid, diarylheptanoid and gingerol biosynthesis | Metabolism | Biosynthesis of other secondary metabolites |
| 15 | map00400 | Phenylalanine, tyrosine and tryptophan biosynthesis | Metabolism | Amino acid metabolism |
| 14 | map00591 | Linoleic acid metabolism | Metabolism | Lipid metabolism |
| 14 | map00906 | Carotenoid biosynthesis | Metabolism | Metabolism of terpenoids and polyketides |
| 13 | map00909 | Sesquiterpenoid and triterpenoid biosynthesis | Metabolism | Metabolism of terpenoids and polyketides |
| 11 | map00902 | Monoterpenoid biosynthesis | Metabolism | Metabolism of terpenoids and polyketides |
| 11 | map00960 | Tropane, piperidine and pyridine alkaloid biosynthesis | Metabolism | Biosynthesis of other secondary metabolites |
| 9 | map00062 | Fatty acid elongation | Metabolism | Lipid metabolism |
| 6 | map00966 | Glucosinolate biosynthesis | Metabolism | Biosynthesis of other secondary metabolites |

Table S5 The information of gene heat map

| Gene_id | WT | PnWRKY9 | Gene description |
| --- | --- | --- | --- |
| gene_24945 | 8.09 | 2.65 | TIFY domain/Divergent CCT motif family protein |
| gene_48999 | 32.59 | 10.43 | TIFY domain/Divergent CCT motif family protein |
| gene_55793 | 28.65 | 9.47 | TIFY domain/Divergent CCT motif family protein |
| gene_71056 | 79.48 | 37.26 | jasmonate-zim-domain protein 1 |
| gene_57078 | 17.46 | 0.92 | jasmonate-zim-domain protein 1 |
| gene_25414 | 45.92 | 21.56 | jasmonate-zim-domain protein 1 |
| gene_6879 | 19.06 | 2.79 | jasmonate-zim-domain protein 1 |
| gene_7494 | 34.71 | 16.46 | MYC2 transcription factor |
| gene_23059 | 30.23 | 12.81 | MYC2 transcription factor |
| gene_39636 | 0.52 | 1.28 | allene oxide synthase |
| gene_76084 | 5.44 | 11.26 | bZIP transcription factor family protein |
| gene_38137 | 7.46 | 1.25 | Pyridoxal phosphate (PLP)-dependent transferases superfamily protein |
| gene_31447 | 3.85 | 1.65 | Amidase family protein |
| gene_19537 | 2.90 | 1.13 | aspartate aminotransferase 3 |
| gene_12807 | 4.30 | 2.20 | Tyrosine transaminase family protein |
| gene_814 | 0.37 | 0.07 | like AUXIN RESISTANT 2 |
| gene_51768 | 3.36 | 1.60 | F-box/RNI-like superfamily protein |
| gene_63469 | 2.54 | 0.70 | indole-3-acetic acid inducible 14 |
| gene_35909 | 0.96 | 0.23 | indole-3-acetic acid inducible 29 |
| gene_11638 | 2.55 | 1.25 | Transcriptional factor B3 family protein / auxin-responsive factor AUX/IAA-related |
| gene_71805 | 30.26 | 13.72 | Auxin-responsive GH3 family protein |
| gene_72826 | 0.27 | 0.04 | Auxin-responsive GH3 family protein |
| gene_82199 | 13.59 | 5.47 | 2-oxoglutarate (2OG) and Fe(II)-dependent oxygenase superfamily protein |
| gene_57521 | 10.31 | 4.06 | 2-oxoglutarate (2OG) and Fe(II)-dependent oxygenase superfamily protein |
| gene_14528 | 3.02 | 0.53 | CHASE domain containing histidine kinase protein |
| gene_3490 | 2.98 | 0.94 | CHASE domain containing histidine kinase protein |
| gene_69609 | 30.15 | 10.47 | response regulator 1 |
| gene_72764 | 4.05 | 0.98 | response regulator 6 |
| gene_28560 | 14.11 | 6.24 | response regulator 9 |
| gene_3663 | 1.76 | 0.38 | response regulator 9 |
| gene_6696 | 3.68 | 1.69 | response regulator 9 |
| gene_25641 | 3.38 | 1.43 | response regulator 9 |
| gene_78410 | 0.74 | 0.06 | response regulator 17 |
| gene_6710 | 0.43 | 0.05 | response regulator 17 |
| gene_55876 | 75.64 | 35.02 | WRKY DNA-binding protein 33 |
| gene_38545 | 17.25 | 3.87 | WRKY DNA-binding protein 33 |
| gene_83844 | 12.71 | 3.74 | WRKY DNA-binding protein 33 |
| gene_54817 | 1.32 | 3.22 | cyclic nucleotide-gated channel 15 |
| gene_71064 | 0.01 | 0.31 | heat shock protein 90.1 |
| gene_26042 | 1.43 | 3.42 | 3-ketoacyl-CoA synthase 2 |
| gene_57536 | 0.45 | 1.15 | NB-ARC domain-containing disease resistance protein |
| gene_30516 | 5.78 | 15.95 | NB-ARC domain-containing disease resistance protein |
| gene_84150 | 3.65 | 7.74 | camphor resistance CrcB family protein |
| gene_988 | 6.00 | 13.05 | pathogenesis-related family protein |
| gene_36979 | 6.08 | 14.38 | plant intracellular ras group-related LRR 4 |
| gene_441 | 3.06 | 6.72 | Leucine-rich repeat (LRR) family protein |
| gene_25394 | 2.09 | 30.21 | Disease resistance protein (TIR-NBS-LRR class) family |
| gene_28794 | 2.49 | 5.52 | disease resistance protein (TIR-NBS-LRR class), putative |
| gene_20114 | 1.32 | 3.01 | disease resistance protein (TIR-NBS-LRR class), putative |
| gene_16863 | 0.23 | 0.99 | Disease resistance protein (TIR-NBS-LRR class) family |
| gene_81396 | 0.11 | 0.40 | Disease resistance protein (CC-NBS-LRR class) family |
| gene_71142 | 57.97 | 219.66 | cytochrome P450, family 83, subfamily B, polypeptide 1 |
| gene_9080 | 1.33 | 8.32 | cytochrome P450, family 83, subfamily B, polypeptide 1 |
| gene_50700 | 0.99 | 4.42 | cytochrome P450, family 82, subfamily G, polypeptide 1 |
| gene_42339 | 0.65 | 2.53 | cytochrome P450, family 82, subfamily C, polypeptide 4 |
| gene_64246 | 4.04 | 8.17 | cytochrome P450, family 81, subfamily K, polypeptide 2 |
| gene_49155 | 1.46 | 3.52 | cytochrome P450, family 78, subfamily A, polypeptide 10 |
| gene_2560 | 1.10 | 2.33 | cytochrome P450, family 78, subfamily A, polypeptide 10 |
| gene_58336 | 0.42 | 1.71 | cytochrome P450, family 72, subfamily A, polypeptide 11 |
| gene_17920 | 16.31 | 39.09 | cytochrome P450, family 71, subfamily A, polypeptide 25 |
| gene_58009 | 0.48 | 1.54 | cytochrome P450, family 704, subfamily A, polypeptide 2 |
| gene_47717 | 1.07 | 9.81 | Cytochrome P450 superfamily protein |
| gene_3434 | 1.07 | 5.40 | Cytochrome P450 superfamily protein |
| gene_21809 | 0.74 | 2.17 | UDP-glucosyl transferase 72E1 |
| gene_47009 | 3.54 | 11.17 | Peroxidase superfamily protein |
| gene_29358 | 1.96 | 4.77 | Peroxidase superfamily protein |
| gene_14429 | 0.05 | 0.44 | Peroxidase superfamily protein |
| gene_47769 | 1.64 | 6.13 | peroxidase 2 |
| gene_19221 | 0.50 | 1.19 | Glycosyl hydrolase family protein |
| gene_54798 | 5.36 | 12.07 | cinnamoyl coa reductase 1 |
| gene_64772 | 1.28 | 2.67 | 2-oxoglutarate (2OG) and Fe(II)-dependent oxygenase superfamily protein |
| gene_1089 | 25.04 | 11.22 | S-adenosyl-L-methionine-dependent methyltransferases superfamily protein |
| gene_17913 | 17.40 | 6.77 | S-adenosyl-L-methionine-dependent methyltransferases superfamily protein |
| gene_31769 | 1.08 | 0.32 | S-adenosyl-L-methionine-dependent methyltransferases superfamily protein |
| gene_80730 | 25.22 | 11.22 | Peroxidase superfamily protein |
| gene_80731 | 3.18 | 0.40 | Peroxidase superfamily protein |
| gene_39154 | 1.89 | 0.28 | Peroxidase superfamily protein |
| gene_76948 | 4.18 | 1.59 | Peroxidase superfamily protein |
| gene_79404 | 8.18 | 1.82 | Peroxidase superfamily protein |
| gene_66387 | 3.85 | 1.55 | Peroxidase superfamily protein |
| gene_80729 | 1.13 | 0.05 | Peroxidase superfamily protein |
| gene_84095 | 3.42 | 1.22 | Peroxidase superfamily protein |
| gene_79349 | 1.72 | 0.44 | Peroxidase superfamily protein |
| gene_9714 | 2.40 | 0.84 | Peroxidase superfamily protein |
| gene_26748 | 0.62 | 0.00 | Peroxidase superfamily protein |
| gene_69241 | 0.91 | 0.18 | Peroxidase superfamily protein |
| gene_78170 | 2.75 | 0.27 | Peroxidase superfamily protein |
| gene_53170 | 0.46 | 0.11 | Peroxidase superfamily protein |
| gene_1446 | 2.11 | 0.81 | Peroxidase superfamily protein |
| gene_77958 | 0.69 | 0.27 | Peroxidase superfamily protein |
| gene_13663 | 31.19 | 15.12 | O-methyltransferase 1 |
| gene_56641 | 12.36 | 6.29 | HXXXD-type acyl-transferase family protein |
| gene_60014 | 15.96 | 8.18 | HXXXD-type acyl-transferase family protein |
| gene_68835 | 3.06 | 1.32 | GroES-like zinc-binding alcohol dehydrogenase family protein |
| gene_9342 | 2.47 | 1.03 | Glycosyl hydrolase family protein |
| gene_9345 | 0.34 | 0.07 | Glycosyl hydrolase family protein |
| gene_27391 | 3.42 | 0.96 | FAD-binding Berberine family protein |
| gene_26685 | 3.07 | 1.19 | FAD-binding Berberine family protein |
| gene_77015 | 0.41 | 0.04 | FAD-binding Berberine family protein |
| gene_21824 | 0.26 | 0.01 | cytochrome P450, family 98, subfamily A, polypeptide 3 |
| gene_73584 | 0.47 | 0.09 | beta-glucosidase 47 |
| gene_22246 | 2.82 | 0.28 | beta glucosidase 17 |
