# Supplementary material for: Panax notoginseng WRKY Transcription Factor 9 Is a Positive Regulator in Responding to Root Rot Pathogen Fusarium solani
Source: Front Plant Sci. 2022 Jul 14;13:930644. doi: 10.3389/fpls.2022.930644 (PMC9331302; doi:10.3389/fpls.2022.930644)
Supplement: Supplementary file 2 [file Data_Sheet_2.docx]

>PPnDEFL1

agatgttcac ctttttaggt cgatgatagg gttttattaa tcaatctttg tttatttgat 60

ctttctctct gatctgtacg tagaaatatg ccatcggtga taaatggcat cacttccctg 120

atttgctgga cttttttaaa aaaaaataaa ggagatgatg ggtgtgaatc tgccgagtct 180

aaggagatgg ttggtcaatt gggtgaccga tcggaacttg ttgtgtgaca aaattgcttc 240

gtccatactg cagagaaagt ttaatttaag tcgatgcact actaaaaaaa acaagaatac 300

atatgacgga atatggaatt aacaagtcat tttgtggcct ttaaagtagc cgccggctgc 360

tacaaaattt caacaatgga ggcgggactc tgattatatg ataaactagt cagcaaaatg 420

ctttcatttt gcagctggcc ggatatatat tatataggga gccgtgccaa ctaattaaat 480

actccattct tgtattgtgt cacgcgtctt cacatctaaa ttaaaaatat aaatcaaaca 540

cggactt 547
